# Gold-nanosphere mitigates osteoporosis through regulating TMAO metabolism in a gut microbiota-dependent manner

**DOI:** 10.1186/s12951-023-01872-9

**Published:** 2023-04-11

**Authors:** Yueqi Chen, Chuan Yang, Qijie Dai, Jiulin Tan, Ce Dou, Fei Luo

**Affiliations:** 1 Department of Orthopedics, Southwest Hospital, Third Military Medical University (Army Medical University), Chongqing, People’s Republic of China; 2 Department of Biomedical Materials Science, Third Military Medical University (Army Medical University), Chongqing, People’s Republic of China

**Keywords:** Gold nanospheres (GNS), Osteoporosis (OP), Gut microbiota, TMAO (trimethylamine N-oxide) metabolism, Anti-inflammation

## Abstract

Osteoporosis (OP) is a metabolic bone disease characterized by decreased bone mass and increased bone fragility. The imbalance of bone homeostasis modulated by osteoclasts and osteoblasts is the most crucial pathological change in osteoporosis. As a novel treatment strategy, nanomedicine has been applied in drug delivery and targeted therapy due to its high efficiency, precision, and fewer side effects. Gold nanospheres (GNS), as a common kind of gold nanoparticles (GNPs), possess significant antimicrobial and anti-inflammatory activity, which have been applied for the treatment of eye diseases and rheumatoid arthritis. However, the effect of GNS on osteoporosis remains elusive. In this study, we found that GNS significantly prevented ovariectomy (OVX)-induced osteoporosis in a gut microbiota-dependent manner. 16S rDNA gene sequencing demonstrated GNS markedly altered the gut microbial diversity and flora composition. In addition, GNS reduced the abundance of TMAO-related metabolites in OVX mice. Low TMAO levels might alleviate the bone loss phenomenon by reducing the inflammation response. Therefore, we investigated the alteration of cytokine profiles in OVX mice. GNS inhibited the release of pro-osteoclastogenic or proinflammatory cytokines including tumor necrosis factor α (TNF-α), interleukin (IL)-6, and granulocyte colony-stimulating factor (G-CSF) in the serum. In conclusion, GNS suppressed estrogen deficiency-induced bone loss by regulating the destroyed homeostasis of gut microbiota so as to reduce its relevant TMAO metabolism and restrain the release of proinflammatory cytokines. These results demonstrated the protective effects of GNS on osteoporosis as a gut microbiota modulator and offered novel insights into the regulation of the “gut–bone” axis.

**Supplementary Information:**

The online version contains supplementary material available at 10.1186/s12951-023-01872-9.

## Introduction

Osteoporosis is a common metabolic skeletal disorder characterized by bone matrix destruction and compromised bone strength, increasing bone fragility and the risk of fracture [1]. In the past decade, osteoporosis has emerged as a public health problem that affects about 200 million people worldwide. More than half of postmenopausal women may acquire osteoporosis-related fractures including vertebral deformity and hip fracture, which are the common causes of mortality in older adults [2]. Postmenopausal osteoporosis and age-related osteoporosis are the most common clinical subtypes of osteoporosis, with pathophysiological changes associated with excess osteoclastogenesis and severe bone erosion [3]. Osteoclasts are differentiated from monocyte/macrophage lineage cells and could participate in regulating bone resorption in bone homeostasis [4]. Current treatment strategies for osteoporosis mainly focus on anti-osteolysis (i.e. estrogens, bisphosphonates) and anabolic agents (i.e. teriparatide, abaloparatide), which have several limitations due to large side effects and lack of evidence of long-term efficacy. Recent studies illustrated that gut microbiota may participate in modulating bone metabolism and the progression of osteoporosis. The composition, structure, and diversity of the intestinal microbial population in osteoporosis patients are significantly changed, including the increase of *Fusobacterium*, *Dialister*, *Faecalibacterium*, and *Tolumonas*, and the decrease of *Bacteroides* and *Roseburia spp*, leading to a state of dysbiosis [5, 6]. Preclinical animal models proved that gut microbiota alterations decrease the quality and strength of bone tissue. The depletion of gut microbiota could negatively modulate the number of osteoclasts and prevent bone erosion [7, 8]. In addition, supplementation of probiotics in the diets of ovariectomy (OVX) mice could reverse the poor progression of osteoporosis. It was also verified in human that gastrointestinal tract flora of normal people is necessary to prevent estrogen deficiency-induced osteoporosis [9, 10]. The abundance of cell signaling molecules and metabolites produced by the gut microbiota may make coupling effects for the “gut-bone” axis. For instance, butyrate produced by gut luminal microbiota mediates the increase of bone marrow (BM) derived regulatory T cells (Tregs) in the anabolic action of PTH and thereby activates bone formation [11]. Trimethylamine-N-oxide (TMAO), a gut microbial-dependent metabolite of dietary choline, has been demonstrated to have a close correlation with multiple diseases such as cardiovascular disease, cancer, and kidney disease [12, 13]. There are several lines of evidence suggesting that TMAO is involved in the maintenance of bone health, but the underlying mechanisms remain unclear.

TMAO is derived from the oxidation of trimethylamine (TMA) by the hepatic flavin monooxygenases (FMO1 and FMO3). The formation of TMAO and TMA is dose-dependent and closely related to the composition of intestinal microflora [14]. TMA is formed by the metabolism of nutrient substrates such as carnitine, phosphatidylcholine/choline, betaine, dimethylglycine, and ergothionine present in the diet. Many bacteria species such as *Clostridia, Proteus, Shigella, and Aerobacter* could produce a large amount of TMA [15]. Generally, changes in diet, dysbiosis of gut microbiota, or impairment of the gut-blood barrier increase TMAO concentration. Recent studies found that high TMAO level is negatively correlated with bone mineral density (BMD) in osteoporosis [16]. D-galactose/sodium nitrite treatment dramatically influences the abundance of *Bifidobacterium* and leads to an abnormal ratio of *Firmicutes/Bacteroidetes*, which increases TMAO levels and ultimately contributes to aging-related osteoporosis [17]. Osteoporosis is a chronic inflammatory disease, in which pro-inflammatory cytokines including interleukin (IL)-1, tumor necrosis factor α (TNF-α), and IL-6 serve as primary mediators of the accelerated bone loss at estrogen deficiency [18, 19]. Many studies suggested that increased levels of pro-inflammatory cytokines such as TNF-α, sTNF-R p75, and IL-1β are positively associated with the concentration of TMAO in plasma, revealing the close connection between TMAO and low-grade inflammation [20–22]. Therefore, reversing intestinal dysbiosis and decreasing TMAO abundance in osteoporosis can potentially mitigate its clinical symptoms. However, few studies have been designed to investigate the effect of TMAO on bone metabolism, as well as the underlying molecular mechanisms.

Nanomaterials have become a novel therapeutic strategy for several serious diseases through their anti-angiogenesis and anti-inflammation activities. Many nanoparticles (NPs) such as silver and titanium dioxide (TiO2) have been proved to affect the gut microbial community by accumulation in the intestine [23–25]. Gold-nanospheres (GNS), with excellent optical properties, biocompatibility, surface adsorbing capacity, and low biotoxicity, are widely applied in cell induction, drug carrier, clinical diagnosis, and antibody labeling. *Saima Hameed et al.* proved that GNS exhibit antimicrobial activity against *Escherichia coli, Pseudomonas aeruginosa*, and *Staphylococcus aureus* [26]. Metagenomic analysis revealed that GNS effectively affect the microbial community structure, enhancing the abundances of *Proteobacteria, Bacteroidetes, and Firmicutes*, and decreasing the abundance of *Actinobacteria* [27]. In addition, GNS exhibit dramatic anti-angiogenic and anti-inflammatory actions, with wide application in clinical trials to inhibit inflammation [28]. Therefore, we hypothesized that GNS could improve intestinal micro-ecological disorders and ameliorate the inflammatory response, thereby alleviating the pathologic symptoms of osteoporosis.

To verify our hypothesis, we have established an OVX-induced osteoporosis mice model and investigated the regulatory function of GNS on the composition of intestinal flora and TMAO metabolism by combining the analysis of microbiome and metabolome. Our results suggested that GNS could alleviate OVX-induced osteoporosis in mice, significantly improve intestinal dysbiosis, and restore the dysregulated TMAO metabolism. Moreover, the decrease in proinflammatory cytokines including TNF-α, IL-6, and G-CSF might be responsible for the protective effect of GNS on osteoporosis. Collectively, these findings proved that intestinal microbiota and microbial TMAO metabolites participated in the protective effect of GNS on osteoporosis treatment. Maintaining the dynamic balance of microbiota-TMAO may be a promising therapeutic strategy for osteoporosis.

## Materials and methods

### Synthesis and characterization of GNS

The GNS used in this experiment were purchased from Wuhan MICE Biotechnology Co. Ltd, and were synthesized by the citrate reduction method. The specific preparation method is provided in the Supplementary Materials and Methods. The GNS sample was dispersed into the water by ultrasonic dispersion, and then put into the potential pool to measure zeta potential by Zetasizer Nano ZS90. In addition, scanning electron microscope (Crossbeam 340, Zeiss) was used to measure the size and homogeneity of the nanoparticles. GNS samples were placed on the carbon-coated copper grid for natural drying and then stained with 2% uranyl acetate. Images were acquired using backscattered electron (BSE) detectors at 10 or 15 kV and 30 Pa. We chose the average nanoparticle size as the size of the sample.

### Animals and treatments

All experimental mice were 6–8-week-old C57BL/6J female mice (weighing 18–22 g), which were taken from the animal center of Army Medical University. All experiments related to these mice abided by the rules of the care and use of laboratory animals and got permission from the Army Medical University (No. AMUWEC20210609). Mice were assigned to six groups: OVX mice (n = 5), OVX mice treated with GNS (n = 5), ABX (OVX) mice (n = 3), ABX(OVX + GNS) mice (n = 5), FMT(OVX) mice (n = 4) and FMT(OVX + GNS) mice (n = 4). GNS were dosed at 0.01 mg/g bw/day, at the stock concentration of 1 mg/ml and a volume of 0.01 ml/g bw over the 56 days. The control groups were treated with an equal volume of normal saline. Animals were dosed between 9 AM and 12 noon.

The Antibiotics (ABX) groups were administered a cocktail of ABX (vancomycin 100 mg/kg; neomycin sulfate 200 mg/kg; metronidazole 200 mg/kg; and ampicillin 200 mg/kg) before OVX surgery for 7 days. Subsequently, the mice received an ABX in the drinking water containing vancomycin (500 mg/L), metronidazole (1 g/L), ampicillin (1 g/L) and neomycin sulfate (1 g/L) throughout the next 8 weeks. The antibiotic-containing water was replaced every other day. The detailed illustration of the procedure of fecal microbiota transplantation (FMT) is in the next section.

### Fecal microbiota transplantation

FMT was conducted in accordance with the improved method that was used previously [29–31]. To be specific, mice accepted ABX treatment (vancomycin 100 mg/kg; neomycin sulfate 200 mg/kg; metronidazole 200 mg/kg; and ampicillin 200 mg/kg) for 7 days to deplete gut microbiota. The OVX surgery was performed subsequently to establish the murine osteoporosis model. During the next 8 weeks, the mice were randomized into two groups, which were referred to as “FMT(OVX + GNS)” and “FMT(OVX)”. The two groups were administered orally with feces (suspended in saline, 200 µl/mouse) derived from OVX + GNS mice (referred to as “OVX + GNS donors”) and OVX mice (referred to as “OVX donors”) every other day respectively. The specific preparation process of fecal suspension is as follows: feces collected from donor mice were pooled and diluted 1:10 (w/v) with saline, and then homogenized for 2 min with vortex to obtain fecal suspension. Subsequently, the fecal suspension was centrifuged at 500×g for 3 min to remove particulate matter. It is important to notice that the supernatant needs to be aspirated under anaerobic conditions and given to mice by gavage within 10 min to prevent changes in fecal flora. All these mice were euthanized on day 56 after OVX surgery, and their femurs were obtained for subsequent analysis. Fecal samples were also collected for further analyses.

### µCT and histological analysis

The Bruker Micro-CT Skyscan 1272 system (Kontich, Belgium) was applied for µCT and the isotropic voxel size was 5.0 μm. The harvested bone tissues were fixed in 4% polyoxymethylene and scanned by a 60 kV X-ray tube (166 µA, 1700 ms integration time). Trabecular bones were thresholded at 86–255 (8-bit gray scale bitmap). The isotropic voxel size used in the whole femur scan is 148 μm and Nrecon (Ver.1.6.10, Kontich, Belgium) was used to reconstruct. The processing and analysis software was CT Analyser (Kontich, Belgium, Ver. 1.15.4.0).

In order to evaluate the side effects of GNS, the tissue samples of the liver and kidney were fixed in 10% formalin overnight. Then the samples were embedded in paraffin and cut into sections for H&E staining.

### Cytokine microarray assay

The serum samples of mice were collected for performing cytokine microarray assay. The specific detailed experiment process is as follows. The beads were shaken at 1400 rpm for 30s, diluted with assay buffer, and shaken again at 1400 rpm for 30s. 50 µl of beads were added to each well of a 96-well plate, and the plate was washed 3 times. Add 50 µl of standard and sample to 96-well plate respectively, cover the plate with parafilm, and shake it at 850 rpm for 30 min in the dark at room temperature. Subsequently, the samples were discarded from the incubated 96-well plate and the plate was washed 3 times. The detection antibody was diluted with antibody diluent according to the instructions. Add 25 µl of diluted detection antibody to each well, then cover the plate with parafilm and shake it at 850 rpm for 30 min at room temperature in the dark. Then, the detection antibody was discarded from the incubated 96-well plate and the plate was washed 3 times. Streptavidin-PE was diluted with antibody diluent according to the instructions. Add 50 µl of diluted streptavidin-PE to each well, put on the parafilm, and shake it at room temperature for 10 min at 850 rpm under dark condition. Wash the plate 3 times and add 125 µl of assay buffer to each well to resuspend beads, the plate was covered with parafilm, and shaken at room temperature for 30s at 850 rpm in the dark. Finally, the Bio Plex 200 machine was used for data acquisition.

### 16S rDNA gene high-throughput sequencing

Firstly, the total DNA of gut microbiota from OVX and OVX + GNS mice was extracted using the E.Z.N.A. ®Stool DNA Kit (D4015, Omega, Inc., USA) according to the manufacturer’s instructions. Nuclear-free water was used for blank. The DNA samples were eluted in 50 µl of Elution buffer and stored at -80 °C until PCR measurement by LC-Bio Technology Co., Ltd (Hangzhou, China). Then we used 2% agarose gel electrophoresis to evaluate the quantity and quality of the PCR products. The PCR products were purified by AMPure XT beads (Beckman Coulter Genomics, Danvers, MA, USA) and quantified by Qubit (Invitrogen, USA). The size of the amplicon pools was assessed on Agilent 2100 Bioanalyzer (Agilent, USA) and the quantity was assessed on the Library Quantification Kit for Illumina (Kapa Biosciences, Woburn, MA, USA). Samples were sequenced on an Illumina NovaSeq platform provided by LC-Bio and the detailed analysis methods have been shown in the Supplementary Materials and Methods.

### Fecal TMAO metabolomics quantitative analysis

Samples comprising 50 mg of feces were added into 1 ml extraction solution that consists of acetonitrile, methanol, and water (2:2:1) with isotopically-labeled internal standard mixture, and then extracted according to the manufacturer’s protocol (Biotree Biomedical Technology Co., Ltd., Shanghai, China). LC-MS/MS analyses were performed using a UHPLC system (Vanquish, Thermo Fisher Scientific) with a UPLC BEH Amide column (2.1 mm × 100 mm, 1.7 μm) coupled to Q Exactive HFX mass spectrometer (Orbitrap MS, Thermo). The detailed methods have been shown in the Supplementary Materials and Methods.

### Integrated microbiome–metabolome analysis

In this study, the interaction between gut microbiota and TMAO-related metabolites was explored by integrated microbiome–metabolome analysis. In this analysis process, spearman correlation analysis was conducted on the differentiated secondary metabolites obtained by metabolomics screening and the significantly differentiated bacterial genera obtained by 16S rDNA sequencing analysis, so as to obtain the relationship among the differentiated metabolites and the differentiated bacterial genera. Based on the calculated results, appropriate screening conditions were selected to obtain the final correlation and network diagram between differential metabolites and differential bacterial genera.

### Enzyme-linked immunosorbent assay (ELISA)

Quantification of serum CTX-1 (C-telopeptide of type I collagen) was investigated using Mouse CTX-1 ELISA Kits (NBP2-69074, Novus, USA), according to the manufacturer’s protocol. Briefly, 100 µl serum was pipetted in a precoated well plate and mixed with 50 µl sample solution, then incubated at 37 ℃ for 1 h. Upon washing with wash solution, we added 50 µl solution A and 50 µl solution B to each well, and read optical density of the mixture using spectrophotometry with 450 nm wavelength. Serum CTX-1 levels were calculated using a standard curve and analyzed by the software Graphpad Prism 7.

### Statistical analysis

In this study, we determined whether there is a significant difference between different groups through appropriate statistical methods that are based on the normality of data and the homogeneity of variance. In the quantification analyses of µCT, the comparisons among different groups were calculated by the student’s t-test (unpaired, two-tailed) using the software Graphpad Prism 7.0, when the F test was not significant. If the F test was significant, we performed the comparisons between different groups by the unpaired t-test with Welch’s correction. Bacterial alpha-diversity and beta-diversity were determined based on the obtained ASV (feature) sequence and ASV abundance table analysis. Alpha-diversity was presented by Chao1, Observed species, Shannon, and Simpson, which were calculated by QIIME2. Beta-diversity was also calculated through QIIME2 and mainly including three kinds of distances (weighted-unifrac, unweighted-unifrac and bray-curtis). The related graphs were drawn by R package. Bacterial taxonomic analyses and comparisons between the two groups were based on the levels of bacterial phylum, class, order, family, and genus, and were performed by Wilcoxon rank-sum test (Filter threshold: p-value < 0.05). Additionally, the linear discriminant analysis (LDA) scores were calculated with the software nsegata-lefse (094f447691f0) based on the normalized relative abundance matrix, which contributes to determining the predominance of bacterial communities between groups. In the process of fecal TMAO metabolomics quantitative analysis, the principal component analysis (PCA) and orthogonal projections to latent structures discriminate analysis (OPLS-DA) were performed in the SIMCA16.0.2 software package (Sartorius Stedim Data Analytics AB, Umea, Sweden). The metabolites with VIP > 1.0 and p < 0.05 (Student’s t-test) were considered to possess significant differences. All data were presented as the mean ± standard error. A *p-value < 0.05 was considered significant and NS represented as no significance. In addition, p<0.01 and p<0.001 were presented as ** and ***.

The completed detailed reagents information and relevant high-Sequencing methods or analytic procedures are provided in the Supplementary Materials and Methods.

## Results and discussion

### The synthesis and characterization of GNS

**Fig. 1:**
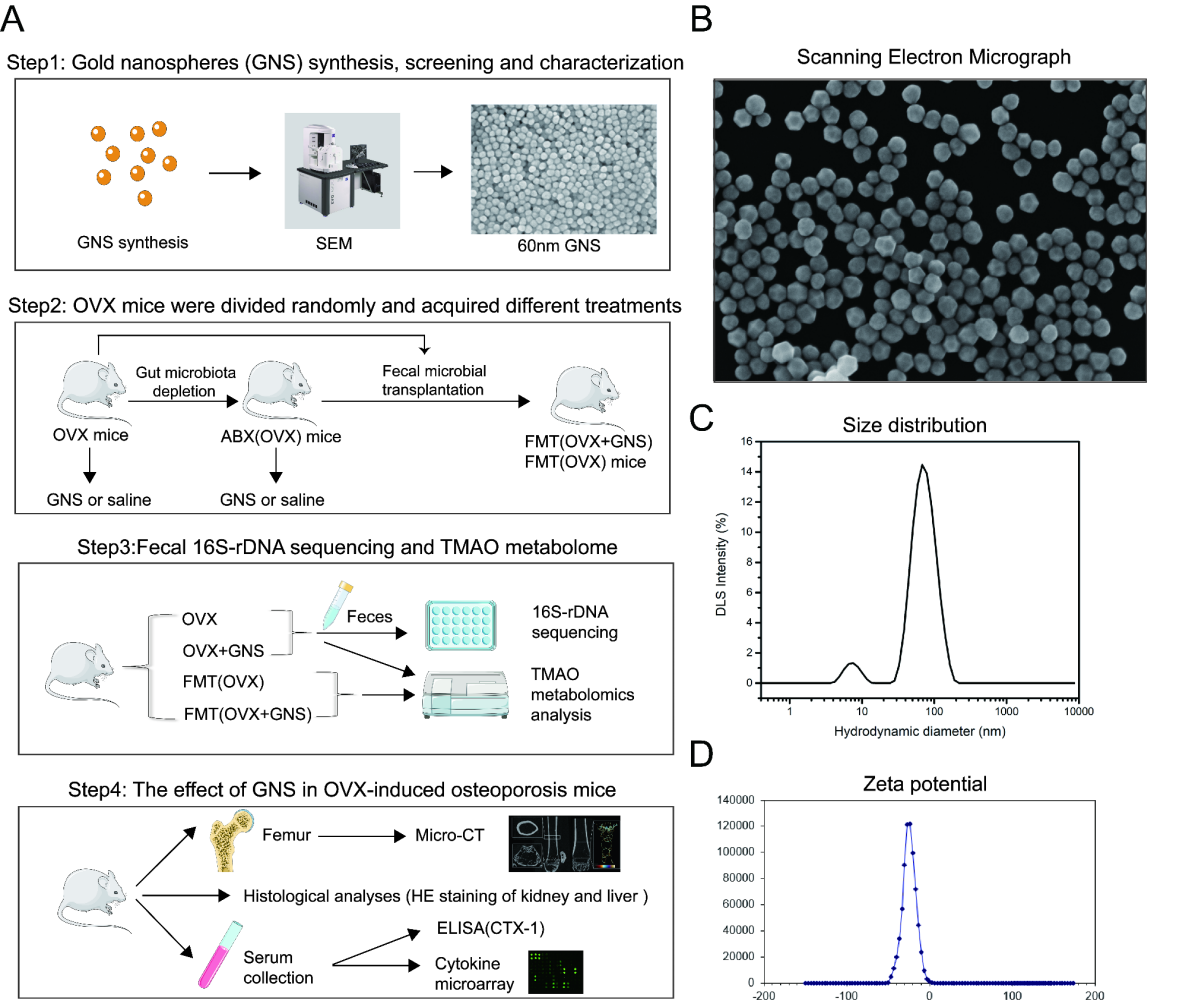
The characterization and main experimental design of GNS. (A) The detailed experimental procedures of the study. (B) The characterization of GNS through scanning electron microscope. (C) Size distribution of GNS. (D) Zeta potential of GNS.

This main experimental design is well shown in Fig. 1A. The detailed chemical synthesis procedure has been described in Supplementary Materials and Methods. SEM was used to measure the sizes and homogeneity of the nanoparticles, and the representative SEM image proved that GNS distributed uniformly and the particle size of the GNS used in this experiment is about 60 nm (Fig. 1B and 1C). The zeta-potential values were approximately − 24.6 mV for GNS (Fig. 1D).

### GNS administration alleviated pathological features of OVX-induced osteoporosis

**Fig. 2:**
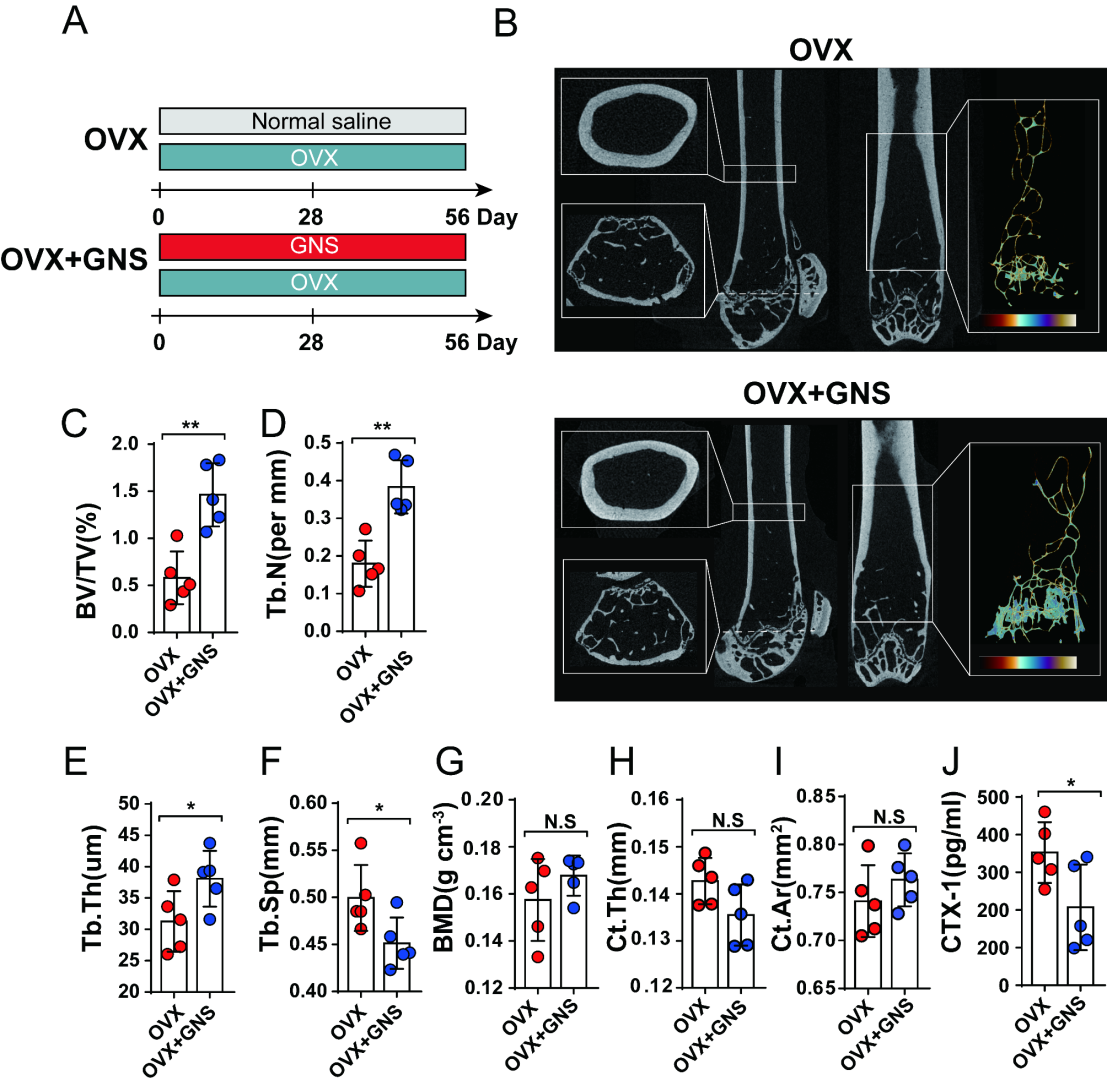
GNS treatment improved OVX-induced experimental osteoporosis. (A) To construct an experimental osteoporosis model and assess the improvement of GNS in osteoporosis, 6–8-week-old female C57BL/6J mice were selected for ovariectomy and randomly assigned to OVX or OVX + GNS group. The OVX and OVX + GNS groups were intragastrically administered by saline and GNS from day 0 to day 56, respectively. (B) Representative µCT images of longitudinal section femurs, cross-sectional view of the distal femurs and reconstructed trabecular structure of the ROI. (C-I) Quantitative analysis of BV/TV, Tb.N, Tb.Th, Tb.Sp, BMD, Ct.Th and Ct.Ar. (J) Quantitative analysis of the concentration of bone turnover marker CTX-1 in serum. (A-J) n = 5 mice.

The OVX-induced murine osteoporosis model has been widely used to simulate the typical clinical phenotypes of osteoporosis, such as bone loss, bone microstructure degeneration, and increased bone fragility [32]. Therefore, the OVX osteoporotic mice were employed to investigate whether GNS has a therapeutic effect on osteoporosis. Mice were separated into two groups: the OVX group (control group) and OVX + GNS (0.01 mg/g) group. The OVX + GNS group was intragastrically administered with GNS suspension from day 0 to day 56, while the control group was treated with normal saline (Fig. 2A). Histological analyses (H&E staining) showed that GNS had no adverse effects on the kidney and liver of mice and possessed good biocompatibility (Supplementary Figure S1A,1B). After euthanization, we contoured dissected femur trabecular and cortical bone for analysis using µCT. The representative µCT images of femurs and reconstructed trabecular structure demonstrated that GNS attenuated bone loss in OVX mice (Fig. 2B). Quantification analyses showed the same tendency. GNS markedly increased bone volume/tissue volume ratio (BV/TV, *p* < 0.01), trabecular number (Tb.N, *p* < 0.01), trabecular thickness (Tb.Th, *p* < 0.05), and reduced trabecular separation (Tb.Sp, *p* < 0.05) (Fig. 2C-2F). Interestingly, there was no significant difference in BMD between the two groups (*p* > 0.05) (Fig. 2G). Additionally, we also tested the related index of cortical bone including cortical bone thickness (Ct. Th, *p* > 0.05) and cortical bone area (Ct.Ar, *p* > 0.05) (Fig. 2H and 2I). Moreover, bone turnover biomarkers (BTMs) are a class of byproducts derived from the process of bone metabolism that could be measured in urine and serum [33]. CTX-1 in serum was known as a bone resorption-relevant biomarker to reflect the progression of bone erosion. Thus, we measured the concentration of CTX-1 in serum and found that GNS markedly decreased the level of CTX-1, further suggesting the protective effect of GNS on bone loss (p < 0.05) (Fig. 2J). Taken together, these results demonstrated that GNS treatment significantly ameliorated OVX-induced osteoporosis in vivo.

### GNS attenuated OVX-induced osteoporosis in a gut microbiota-dependent manner

**Fig. 3:**
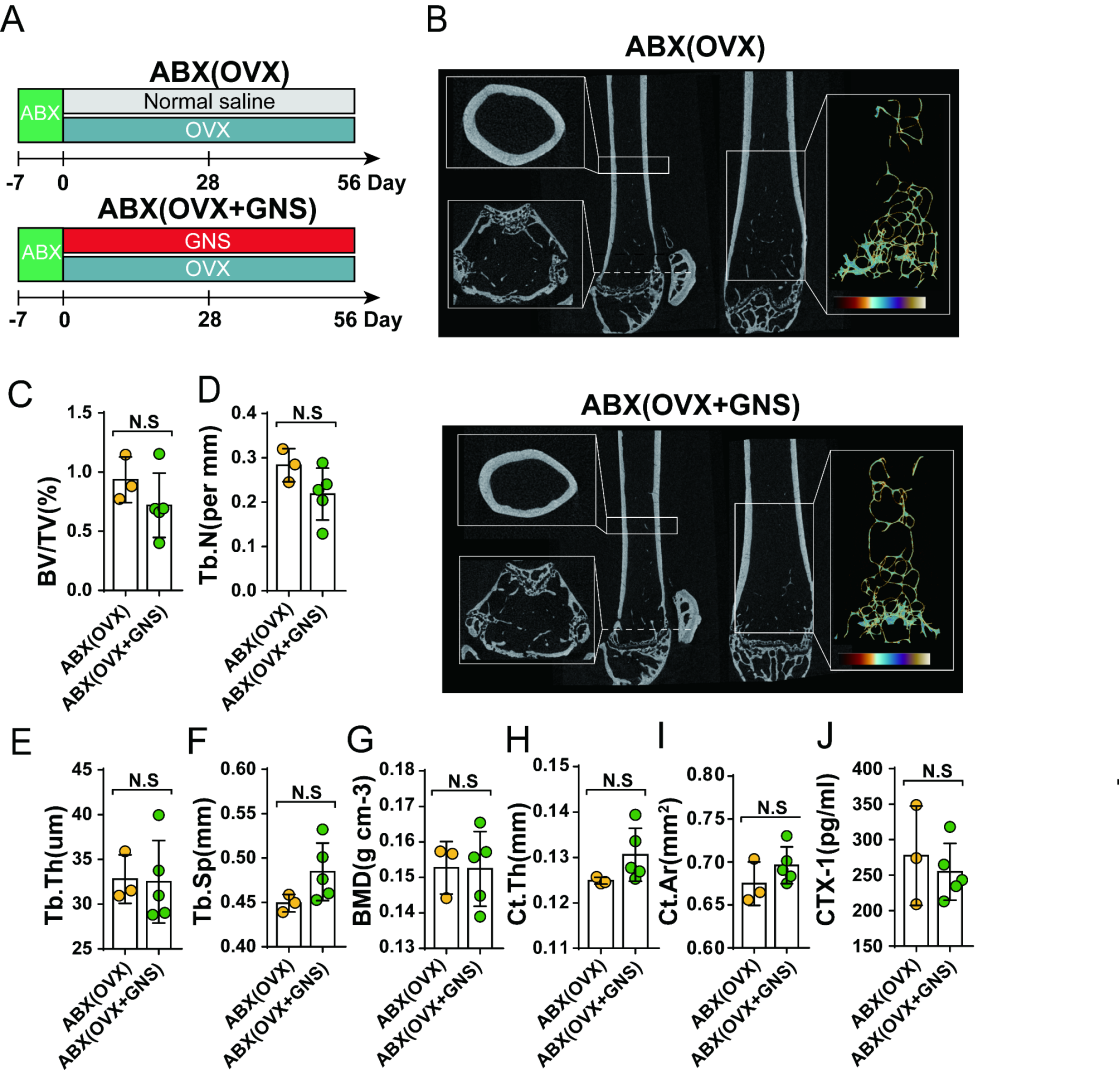
The protective effect of GNS against OVX-induced osteoporosis disappeared after gut-microbiota depletion. (A) The schematic diagram of gut microbiota depletion experiments. (B) Representative µCT images of longitudinal section femurs, cross-sectional view of the distal femurs and reconstructed trabecular structure of the ROI. (C-I) Quantitative analysis of BV/TV, Tb.N, Tb.Th, Tb.Sp, BMD, Ct.Th and Ct.Ar. (J) Quantitative analysis of the concentration of bone turnover marker CTX-1 in serum. (A-J) n = 3 and 5 mice per group.

Although the pathophysiology of osteoporosis is complex and not fully understood, multiple pieces of evidence have proved that the gut microbiota could make effects on the progression of osteoporosis through many different manners such as osteoclastogenesis, intestinal calcium absorption, and so on. To verify whether the protective effect of GNS on OVX-induced osteoporosis is linked to gut microbiota, we have conducted a pseudo-germ-free (PGF) murine model using the quadruple antibiotic cocktails (vancomycin 100 mg/kg; neomycin sulfate 200 mg/kg; metronidazole 200 mg/kg; and ampicillin 200 mg/kg) before GNS administration (Fig. 3A). As shown in Fig. 3B, the µCT images of the ABX(OVX) group were almost indistinguishable from the ABX(OVX + GNS) group. The beneficial effects of GNS on osteoporosis including the alteration of BV/TV, Tb.N, Tb.Th, Tb.Sp, and CTX-1 were completely abolished in the absence of gut bacteria (Fig. 3C-3J).

**Fig. 4:**
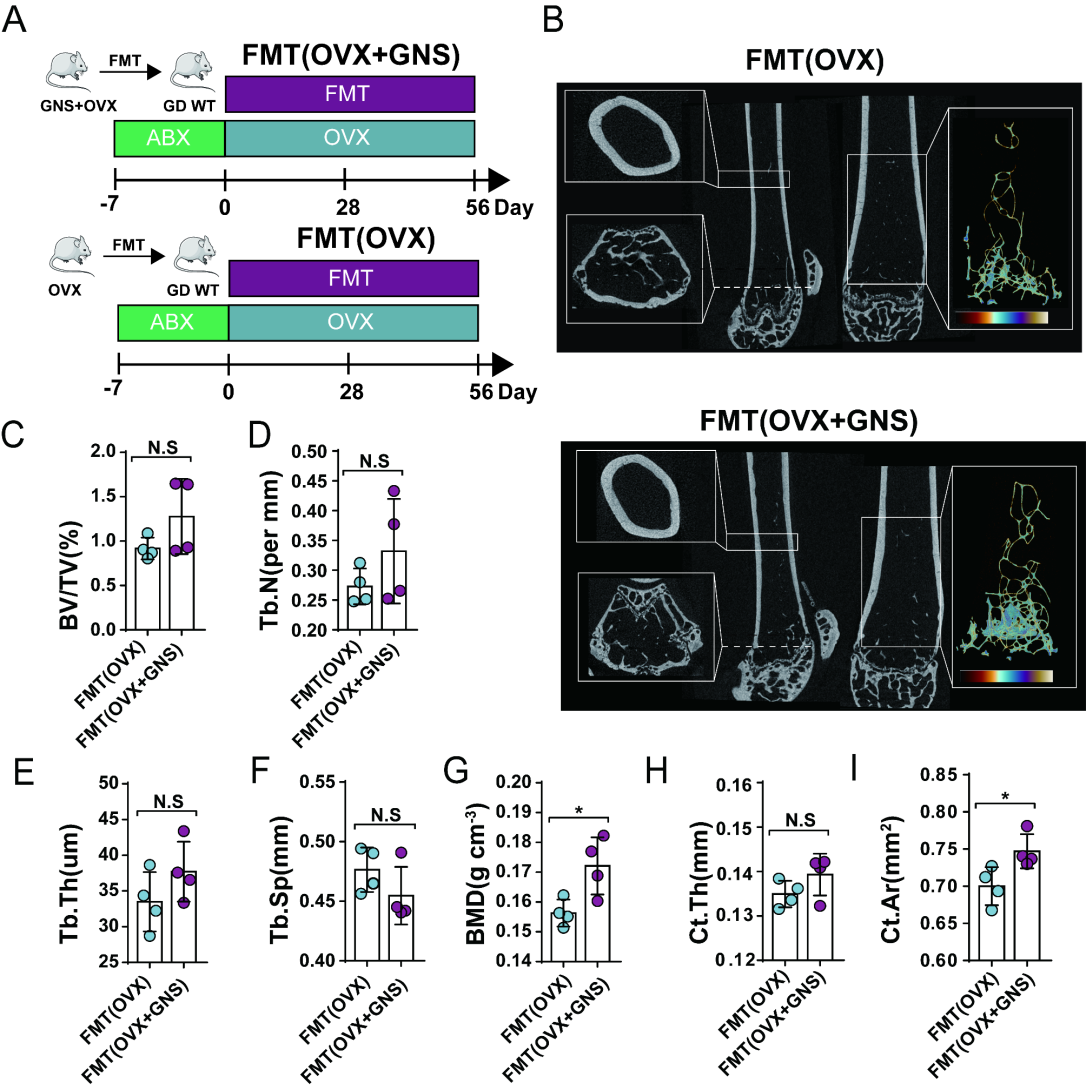
Fecal microbiota transplantation alleviated OVX-induced experimental osteoporosis. (A) The schematic diagram of the FMT experiments. (B) Representative µCT images of longitudinal section femurs, cross-sectional view of the distal femurs and reconstructed trabecular structure of the ROI. (C-I) Quantitative analysis of BV/TV, Tb.N, Tb.Th, Tb.Sp, BMD, Ct.Th and Ct.Ar. (A-I) n = 4 mice per group.

To further confirm whether GNS attenuated OVX-induced osteoporosis in a gut microbiota-dependent manner, we transplanted the microbiota of OVX and OVX + GNS groups into gut microbiota-depleted wild-type (GD WT) mice by gavage every other day as mentioned above (Fig. 4A). BMD and Ct.Ar were markedly increased in FMT(OVX + GNS) group compared with FMT(OVX) group (Fig. 4G and 4I), but other index and µCT images displayed no significant differences between the two groups (Fig. 4B-4F, 4H). These non-significant difference results may be due to the loss of gut microbiota during FM transplantation experiments. All indicators showed the same tendency towards the direction of osteoporosis remission, but the difference was not significant. These findings demonstrated that GNS attenuated OVX-induced osteoporosis in a gut microbiota-dependent manner.

### GNS treatment markedly altered the diversity and composition of gut microbiota

**Fig. 5:**
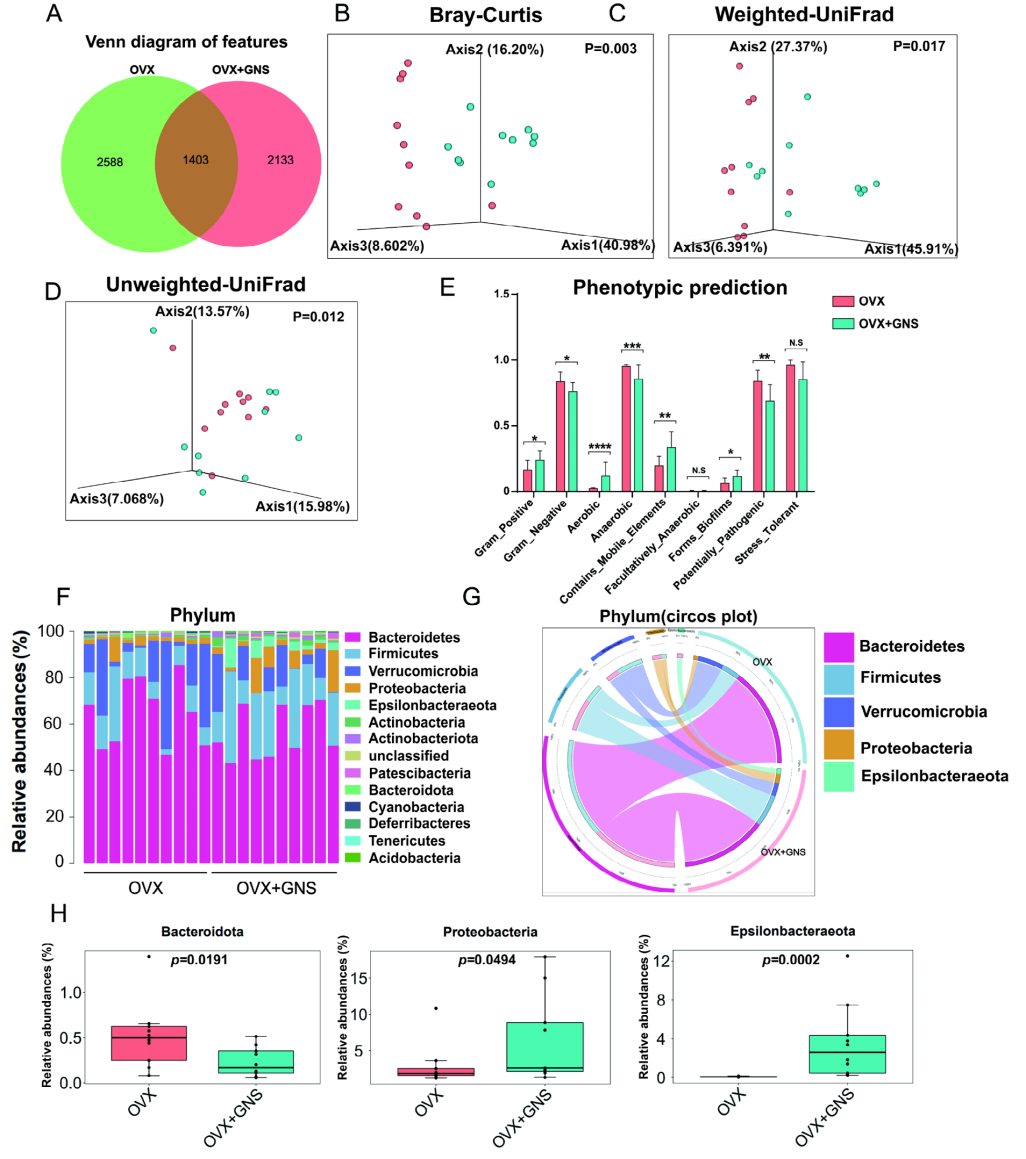
GNS administration markedly altered the gut microbiota diversity and composition. (A) Venn diagram showed the overlapping features in OVX and OVX + GNS groups. (B) Multiple sample PCoA based on the Bray-Curtis distance of beta diversity. (C) PCoA of the Weighted-UniFrad distance. (D) PCoA of the Unweighted-UniFrad distance. (E) Bar graph showing the abundance of microbiota with functional clustering in the OVX and OVX + GNS groups. (F) Bar graphs of the gut microbiota at phylum taxonomic level in OVX and OVX + GNS group. (G) The circos plot showing the relative abundance of bacterial phyla between the OVX and OVX + GNS groups. The different colored ribbon represents specific phylum and the width of ribbon is directly proportional to the abundance of phylum. The ribbon connects bacterial taxa to their respective sample. (H) Relative abundance of *Bacteroidota*, *Proteobacteria*, and *Epsilonbacteraeota* in each group were displayed by bar plots. (A-H) n = 10 samples per group.

Since gut dysbiosis was a pathological factor of osteoporosis and GNS attenuated osteoporosis in a gut microbiota-dependent manner, we investigated the regulatory effects of GNS on the diversity and composition of gut microbiota using 16S rDNA gene sequencing analysis for fecal bacteria. As shown in Fig. 5A, a total of 6124 features were obtained from the feces of OVX and OVX + GNS groups, of which 1403 features were commonly shared in both two groups. These features included 277 species, 205 genera, 68 families, 36 orders, 23 classes, and 14 phyla. The species diversity and differences of bacteria between groups were elucidated using species annotations and the above features.

We originally investigated the beta diversity between the two groups, which could effectively reflect the composition and structure of gut microbiota. The principal coordinate analysis (PCoA) based on the Bray-Curtis distance (p = 0.003), Weighted-UniFrac distance (p = 0.017), and Unweighted-UniFrac distance algorithms (p = 0.012) revealed clearly separated clusters between the two groups, suggesting the different composition of microbiota in OVX + GNS and OVX groups (Fig. 5B-5D). Subsequently, we measured gut microbial-associated alpha diversity using a generalized linear model through different methodologies. The rarefaction curves tended to be flat, which demonstrated that the test can effectively reflect the information of gut microbiota in samples (Supplementary Figure S2A). These alpha diversity indexes including Chao1, Observed species, Shannon and Simpson showed no statistical difference (p > 0.05), suggesting the species richness of intestinal flora was similar between OVX and OVX + GNS groups (Supplementary Figure S2B). In addition, phenotypic prediction showed that gram-positive bacteria, aerobic bacteria, mobile element-containing bacteria enriched, gram-negative bacteria, anaerobic bacteria and potentially pathogenic bacteria were decreased in the OVX + GNS mice (Fig. 5E). To investigate the specific changes in gut microbiota between the two groups, we evaluated the relative abundance of taxa in each group at different levels. As shown in Fig. 5F and  5G, *Bacteroidetes* and *Firmicutes* occupied a relatively high proportion in nearly all samples at the phylum level. As the leading phylum, the proportion of *Bacteroidetes* in the gut flora of OVX and OVX + GNS groups was 64.97% and 56.27%, respectively (*p* = 0.11) (Supplementary Figure S2C). In addition, *Firmicutes* was the second most dominating phylum that accounted for 12.12% and 21.11% in the two groups respectively (*p* = 0.07) (Supplementary Figure S2D). Interestingly, the ratio of *Firmicutes* to *Bacteroidetes* (F/B) in the OVX + GNS group was significantly higher than that in the OVX groups (*p* < 0.05), indicating a higher sensitivity of *Bacteroidetes* to GNS (Supplementary Figure S2E). Furthermore, the differentiated-abundance analysis showed that GNS administration significantly decreased the level of *Bacteroidota* (*p* = 0.02) but increased the level of *proteobacteria* (*p* = 0.05) and *Epsilonbacteraeota* (*p* = 0.0002) (Fig. 5H).

At the class level, we identified a tendency for the *Bacilli* (*p* = 0.023), *Campylobacteria* (*p* = 0.0002) and *Coriobacteriia* (*p* = 0.013) classes to increase in abundance after GNS treatment (Supplementary Figure S3A-3D). In addition, we found that GNS administration significantly increased the levels of *Campylobacterales* (*p* = 0.0002), *Lactobacillales* (*p* = 0.023), *Anaeroplasmatales* (*p* = 0.032), and *Coriobacteriales* (*p* = 0.01) at the order level (Supplementary Figure S4A-4E). As for the family level, the *Lactobacillaceae* (*p* = 0.0233), *Helicobacteraceae* (*p* = 0.0002), *Atopobiaceae* (*p* = 0.0413), and *Marinifilaceae* families (*p* = 0.019) were enriched, while the *Prevotellaceae* family (*p* = 0.0052) was decreased in the OVX + GNS mice (Supplementary Figure S5A-5 F).

**Fig. 6:**
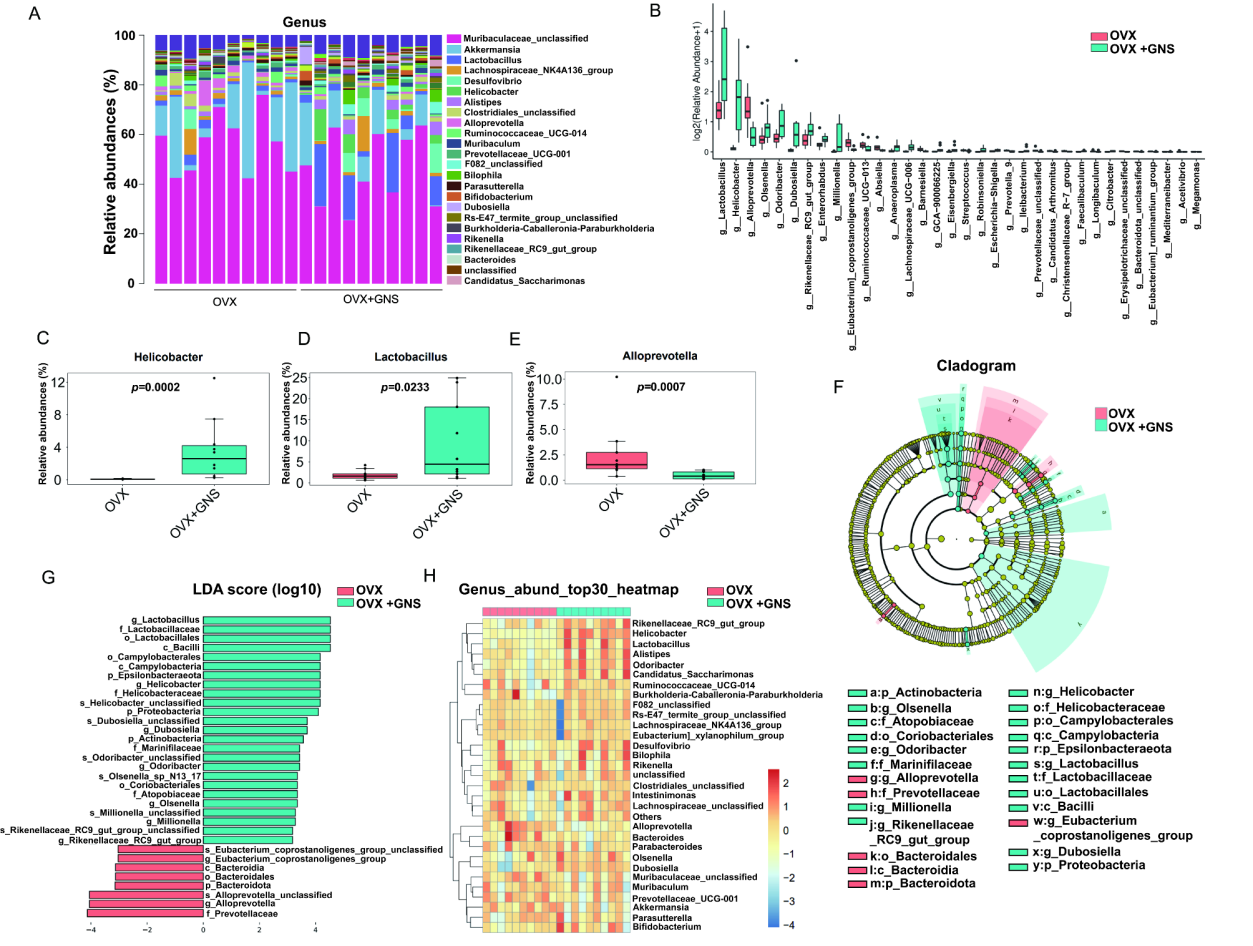
Fecal microbiota composition of OVX and OVX + GNS groups at genus taxonomic level. (A) Bar graphs of the genus taxonomic levels in OVX and OVX + GNS groups. Relative abundance is plotted for each sample. (B) Bar plots comparing taxonomic composition at the genus level. All bacterial genera displayed significant differences between OVX and OVX + GNS groups. (C) Relative abundance of genus *Helicobacter* in each group was displayed by bar plots. (D) Relative abundance of genus *Lactobacillus* in each group was displayed by bar plots. (E) Relative abundance of genus *Alloprevotella* in each group was displayed by bar plots. (F) Taxonomic cladogram based on LEfSe represents the taxonomic association between microbiome communities from OVX and OVX + GNS groups. Yellow nodes displayed no significant difference in species between the two groups. Blue nodes represent the taxonomic types that are enriched in the GNS + OVX group. Species with high abundance in the OVX group were colored in red. (G) LEfSe score illustrated the statistical difference in species between the two groups. (H) Heatmap of selected most differentially abundant features at the genus level. The blue, white and red color represents less, intermediate and high abundance, respectively. (A-F) n = 10 samples per group.

We further analyzed species distributions at genus level and found the two groups showed markedly different compositions and structures in the gut microbiota (Fig. 6A and 6B). The genus *Helicobacter* (0.08% vs. 3.58%, *p* < 0.001) and *Lactobacillus* (1.88% vs. 9.49%, *p* < 0.05) occupied comparatively higher proportion in GNS-treated mice, and the genus *Alloprevotella* (2.57% vs. 0.46%, *p* < 0.001) displayed less abundance in the OVX + GNS group (Fig. 6C-6E). To confirm which kind of bacterium was changed after administration with GNS and played an important role in the progression of OVX-induced osteoporosis, we conducted linear discriminant analysis (LDA) effect size (LEfSe) and Cladogram (based on maximum relative abundance difference in each level) to investigate significant differences in bacterial communities that occupied the leading status between OVX and OVX + GNS groups. The analysis results demonstrated that the family of *Prevotellaceae* (including the subordinate genus *Alloprevotella*), the phylum of *Bacteroidota* (from the class of *Bacteroidia* to the order of *Bacteroidales*), and the genus of *Eubacterium_coprostanoligenes_group* were the important types of bacteria that promote enteric dysbacteriosis in the OVX group. In addition, the order of *Lactobacillales* (including the family of *Lactobacillaceae* and the genus of *Lactobacillus*), the family of *Helicobacteraceae* (including the genus *Helicobacter*), the order of *Coriobacteriales* (including the family of *Atopobiaceae* and the genus of *Olsenella*), the family of *Marinifilaceae* (including the genus of *Odoribacter*), and the phylum of *Actinobacteria* and *Proteobacteria* showed a relatively higher abundance in the OVX + GNS group, which may serve as the crucial types of bacteria with the GNS-mediated alleviation of osteoporosis (Fig. 6F). As for the specific scores, the genus of *Lactobacillus* possessed the highest LDA score of 4.53 (*p* = 0.02), followed by *Helicobacter* with an LDA score of 4.17 (*p* = 0.00015) in the OVX + GNS group. In the OVX group, the genus of *Alloprevotella* had the highest LDA score of 4.06 (*p* = 0.0007), followed by *Bacteroidales* (LDA score = 3.13; *p* = 0.02) and *Eubacterium_coprostanoligenes_group* (LDA score = 3.03; *p* = 0.007) (Fig. 6G). As shown in the heatmap, the genus *Lactobacillus* and *Helicobacter* were markedly enriched in the OVX + GNS group, while the genus *Alloprevotella* and *Bacteroidales* accounted for a higher proportion in the OVX group, which was in accordance with the LEfSe analysis results (Fig. 6H). In conclusion, GNS treatment affected the diversity and composition of the gut flora.

### GNS treatment decreased the abundance of metabolite TMAO

**Fig. 7:**
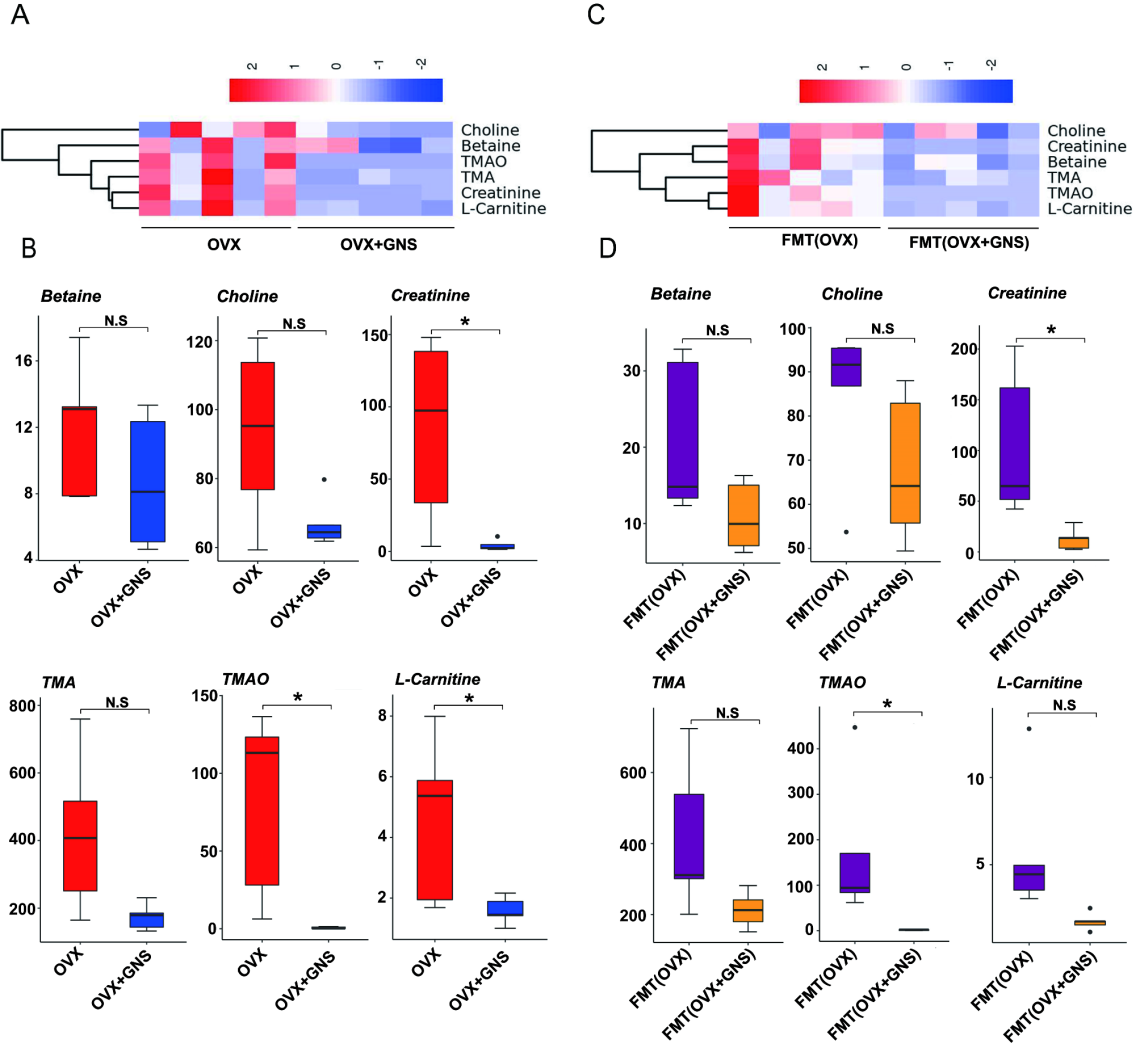
GNS administration decreased the production of TMAO-related metabolites derived from gut microbiota. (A) Heatmap of TMAO-related metabolites between OVX and OVX + GNS groups. The blue, white and red color represents less, intermediate and high abundance, respectively. (B) The concentration of TMAO-related metabolites including betaine, choline, creatinine, TMA, TMAO and L-carnitine from feces between OVX and OVX + GNS groups. (C) Heatmap of TMAO-related metabolites between FMT(OVX) and FMT (OVX + GNS) groups. (D) The concentration of TMAO-related metabolites including betaine, choline, creatinine, TMA, TMAO and L-carnitine from feces between the FMT(OVX) and FMT(OVX + GNS) group. (A-D) n = 5 mice per group.

Previous analysis results of 16S rDNA gene sequencing demonstrated that GNS significantly increased the levels of the genus *Lactobacillus* and reduced the abundance of *Alloprevotella* and *Bacteroidales*, which were related to TMAO abundance[34–36]. As mentioned above, gut microbiota metabolite plays a crucial part in the maintenance of bone homeostasis and high TMAO level has been proved to be associated with bone loss in osteoporosis patients. To explore whether the alteration of gut microbiota influenced the production of TMAO, we performed metabolomics analysis to identify the abundance of TMAO-related metabolites including betaine, choline, creatinine, carnitine, TMA, and TMAO. Consistent with the alterations in microbial diversity and composition, the OVX + GNS and OVX groups had different TMAO profiles. Orthogonal partial least-squares discriminant analysis (OPLS-DA) showed distinct clustering of TMAO between two groups (Supplementary Figure S6A). Principal component analysis (PCA) showed the same tendency (Supplementary Figure S6B). Among these metabolites, TMAO precursor creatinine (*p* = 0.048), L-carnitine (*p* = 0.041), and TMAO (*p* = 0.016) manifested lower amounts in the OVX + GNS group. Although betaine, choline and TMA displayed no significant differences between the two groups, total TMAO-related metabolites in the OVX + GNS group were significantly decreased compared with the OVX group in feces (Fig. 7A and B). To determine whether changes in the levels of TMAO were closely associated with GNS treatment-induced gut flora alterations, we performed targeted TMAO quantitative analysis between FMT groups. Sure enough, the OPLS-DA and PCA revealed clearly separated clusters between the two groups (Supplementary Figure S6C-6D). The production of the TMAO-related metabolites in the FMT (OVX + GNS) was also significantly lower than that in FMT (OVX) group (Fig. 7C). The concentrations of creatinine (*p* = 0.023), and TMAO (*p* = 0.045) in the FMT (OVX + GNS) group were markedly lower than those in the FMT (OVX) group. Although betaine,  choline, L-carnitine and TMA showed no significant differences between the two groups, total TMAO-related metabolites in the FMT (OVX + GNS) group were markedly lower than those in the FMT (OVX) group (Fig. 7D). In conclusion, GNS treatment decreased metabolite TMAO abundance by regulating the diversity, community structure and composition of gut microbiota.

To better determine the connection between gut microbiota and metabolite TMAO, we performed an integrated microbiome-metabolome analysis to find the specific microbiota genera that exhibited significant correlations with TMAO-related metabolites. As is shown in the heatmap of association analysis, 17 genera showed significant correlations with TMAO-related metabolites. Among these genera, 12 genera were positively associated with TMAO-related metabolites and 5 genera displayed a negative correlation. We identified 5 genera of *Absiella*, *Prevotellaceae_unclassified*, *Erysipelotrichaceae_unclassified*, *Candidatus_Arthromitus*, and *Barnesiella* that were marked positively correlated with the level of TMAO-related metabolites by combining the flora abundance (mean abundance > 0.01%) and different distribution (*p* < 0.05) between OVX and OVX + GNS groups. The genera *Prevotellaceae_unclassified*, *Erysipelotrichaceae_unclassified*, *Candidatus_Arthromitus*, and *Barnesiella* were positively correlated with TMAO level, and *Absiella* was *a* positive correlation with the choline level. Indeed, the genera of *Mediterraneibater*, *Acetivibrio*, and *Eubacterium]_ruminantium_group* were also widely positively associated with TMA and creatinine, but their effect on TMAO-related metabolism may be little due to their low abundance (mean abundance < 0.01%).

**Fig. 8:**
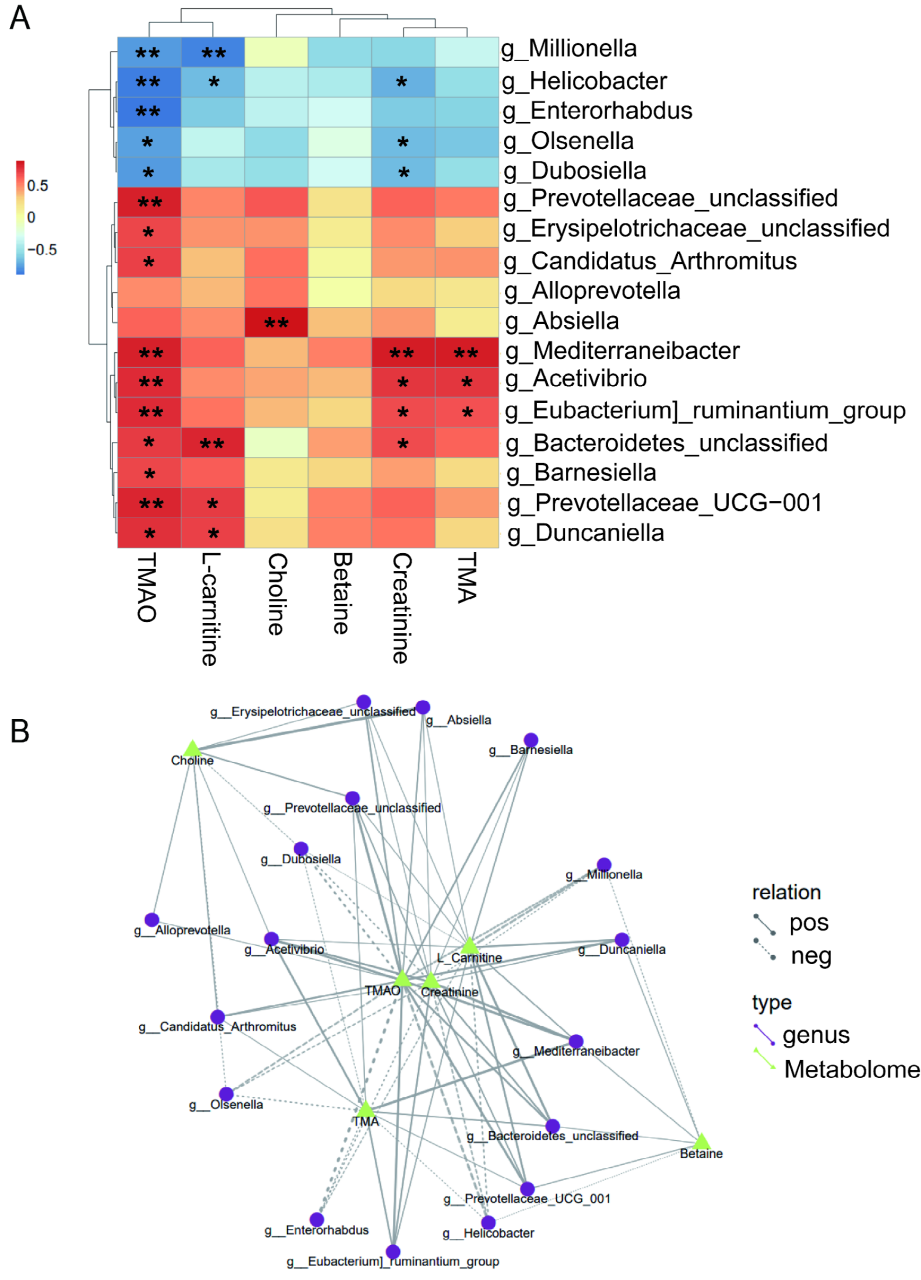
Integrated microbiome–metabolome analysis linked gut microbially produced TMAO with specific bacterial genera. (A) Association heatmap of the 6 TMAO-related metabolites with bacterial genera. Colorings represent the median Spearman correlation coefficient between the 6 TMAO-related metabolites and the indicated gut microbial genera, where the differences are denoted: *, p < 0.05; **, p < 0.01. (B) Association net diagram of TMAO-metabolites with 17 bacterial genera. Different nodes in the figure represent different bacterial genera or metabolites. The shape of bacterial genera is round, and the shape of metabolites is triangular. The lines between the bacterial genera and the metabolites represent the correlation, in which the solid lines represent the positive correlation and the dashed lines represent the negative correlation.

In addition, the genera of *Millionella*, *Helicobacter*, *Enterorhabdus*, *Olsenella*, and *Dubosiella* had a remarkable negative relation with TMAO (Fig. 8A). The last 4 genera may display a stronger inhibitory effect on the production of TMAO-related metabolites due to their higher abundance (> 0.01%). We noted that nearly all these genera were significantly associated with the TMAO level, perhaps due to the fact that TMAO is an end product of metabolism. To further display the correlation between bacterial genus and TMAO-related metabolites, we constructed an association network according to all association rules of 17 genera and TMAO metabolites (Fig. 8B). In conclusion, these results provided suitable information on the association among gut microbiota and TMAO metabolites, which were helpful for further investigation in vitro.

### GNS restrained the release of proinflammatory cytokines to attenuate OVX-induced osteoporosis

**Fig. 9:**
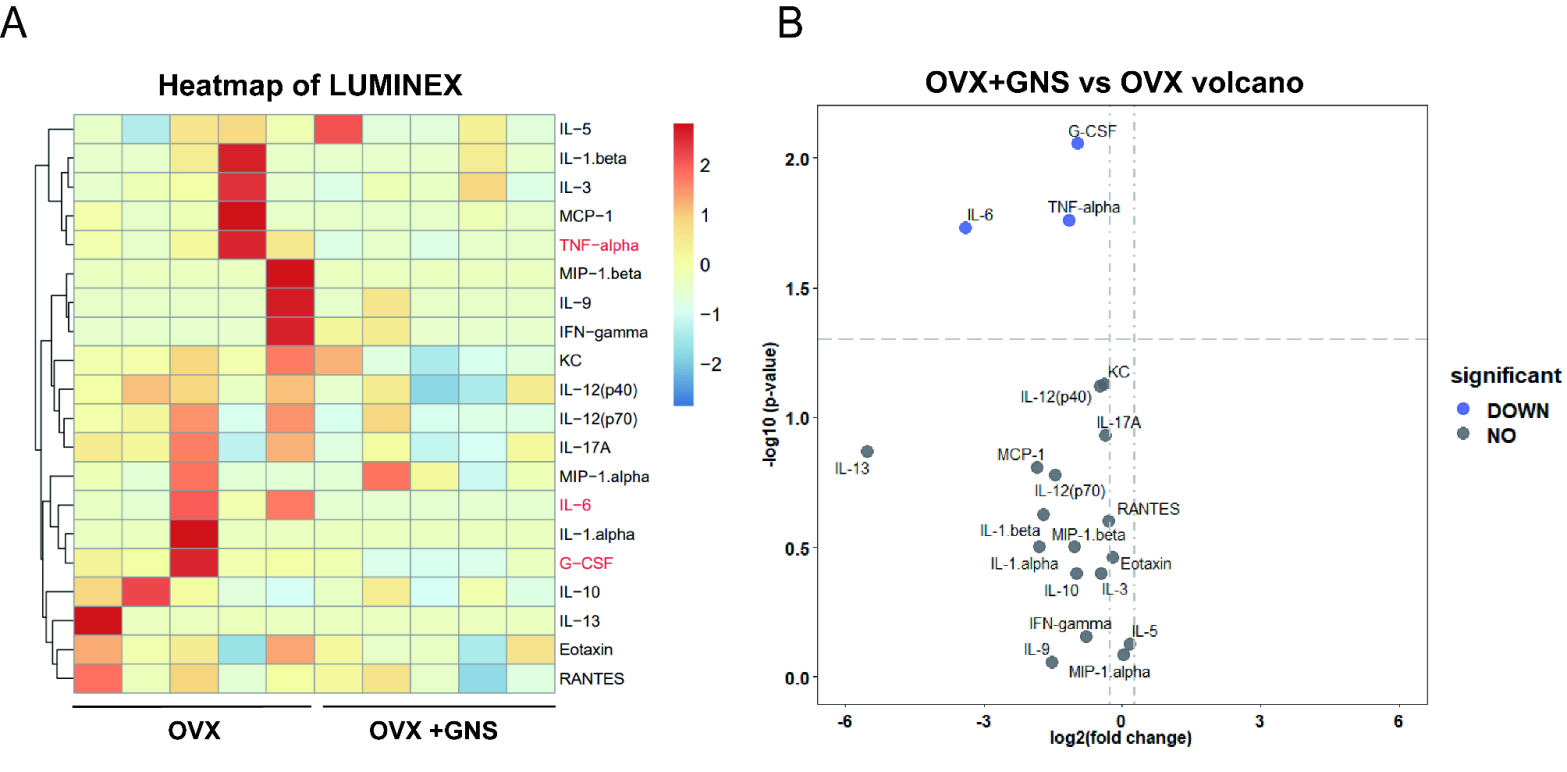
GNS treatment significantly altered the inflammatory cytokine profiles in OVX mice. (A) Heatmap representation of cytokine levels from serum as measured by Luminex. (OVX group, n = 5; OVX + GNS group, n = 5). (B) Volcano plot of cytokine alteration at serum as measured by Luminex bead-based immunoassays.

**Fig. 10:**
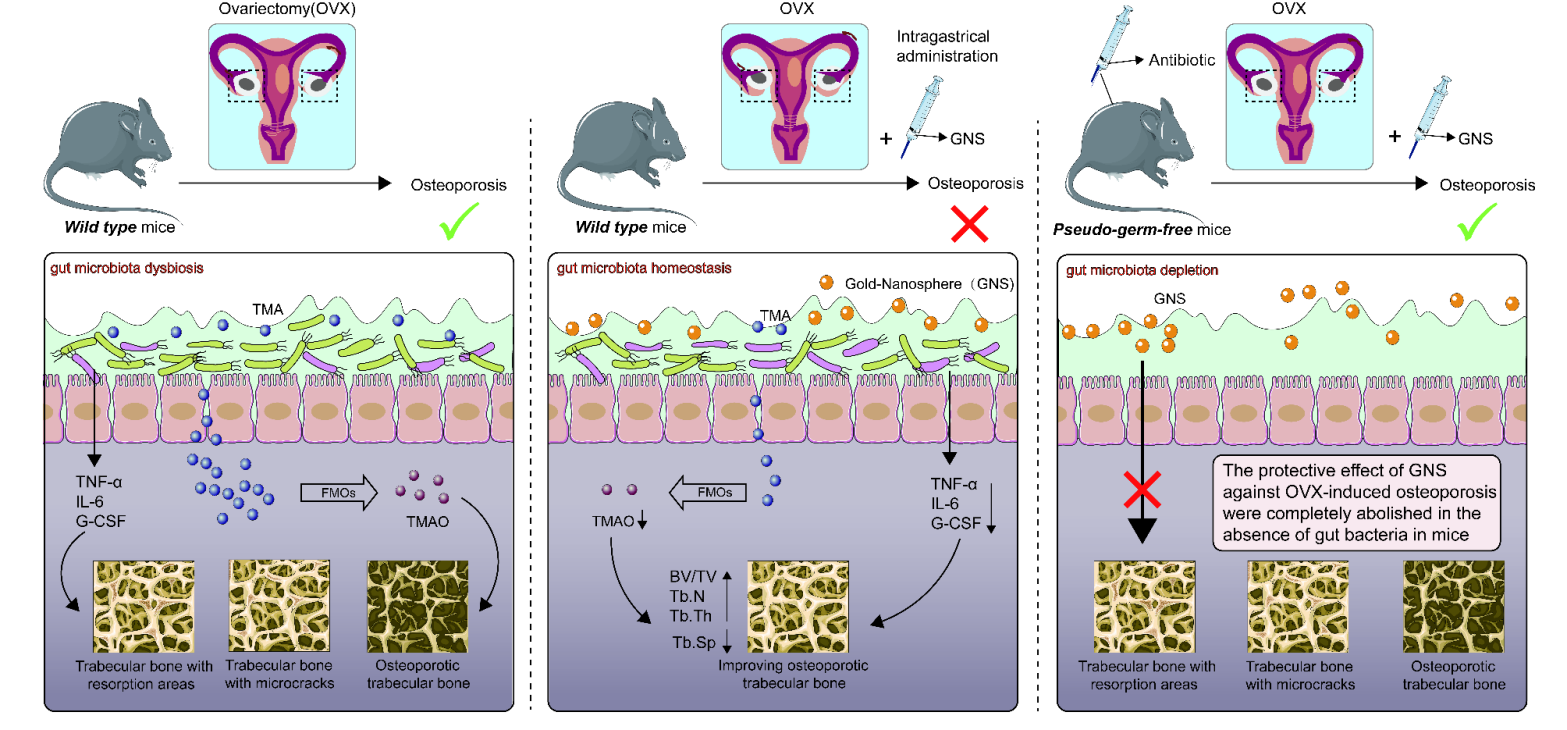
Schematic for our proposed mechanism of the protective effects of GNS on OVX-induced osteoporosis via modulating microbiota homeostasis and relevant TMAO metabolism. Mechanistically, administration with GNS could influence dynamic balance of gut microbiota and negatively regulate the TMAO production to reduce the release of inflammatory or pre-osteoclastic cytokines in serum

As mentioned above, osteoporosis is a chronic inflammatory disease, in which proinflammatory cytokines such as IL-1, IL-6 and TNF-α accelerate bone loss by promoting osteoclast differentiation and activation and inhibiting osteoblast differentiation [37–39]. In addition, TMAO level has been proved to be associated with increased levels of pro-inflammatory cytokines. Therefore, we performed a Luminex cytokine microarray assay to explore whether GNS induced the alteration of different cytokines in serum. As shown in the heatmap, the pro-inflammatory cytokines IL-6, TNF-α, and G-CSF were markedly reduced in the OVX + GNS group compared with the OVX group (p < 0.05) (Fig. 9A). The volcano plot showed that the GNS-induced differentially expressed cytokines (DECs) were consistent with the heatmap (Fig. 9B). In conclusion, GNS administration markedly downregulated the release of pro-inflammatory cytokines, which may be related to the reduction of TMAO (Fig. 10).

## Conclusion

Over 200 million people suffer from osteoporosis, which has become a severe public health challenge worldwide and desiderates novel and effective drugs [40]. In this study, we found that GNS could markedly alleviate the pathological features of OVX-induced osteoporosis in a gut microbiota-dependent manner. Administration of GNS regulated the composition and diversity of osteoporosis-related gut microbiota and reduced the abundance of TMAO-related metabolites, which further decreased the level of proinflammatory cytokines in bone metabolism and ultimately improved the symptoms of osteoporosis. FMT was also performed to prove this gut flora-dependent mechanism. Collectively, our results proved that GNS could effectively improve osteoporosis through gut microbiota modulation, which may serve as a novel and promising drug.

The OVX mice model is the most common animal model of osteoporosis due to its simplicity of operator and phenotype similar to postmenopausal osteoporosis in humans [41]. Several studies have proved that OVX leads to alteration of gut microbiota, including lower abundance in *Verrucomicrobia* and *Deferribacteres*, and higher abundance in *Candidatus Saccharibacteria* and *Tenericutes* [42]. Therefore, the OVX mouse is an ideal model for the study of pharmacological strategies targeting intestinal microbiota disorders in osteoporosis. To ensure the typical phenotype of OVX-induced osteoporosis, we extended the construction time of the entire model and performed µCT analysis at 8 weeks postoperatively. As a novel nanophase material, GNS manifest a regulatory function on bacterial flora in vitro, but the anti-bacterial effects in vivo especially in gut microbiota have rarely been investigated. Therefore, we originally studied the therapeutic potential of GNS in the management of osteoporosis in vivo. As expected, GNS treatment altered the diversity and composition of gut microbiota in the OVX-induced osteoporosis mice, and the depletion of gut microbiota could completely remove the improvement of GNS on the symptoms of osteoporosis, suggesting that the gut microbiota played an important role in the therapeutic effects of GNS.

Recent studies have shown that the diversity of microbiota is decreased in osteoporotic patients, resulting in a state of dysbiosis [5]. Preclinical animal models proved that microbiota dysbiosis can influence bone metabolism, which decreases the quality and hence the strength of bone tissue [7]. Indeed, the gut microbiota includes hundreds of bacterial genera that enhance the ability to extract energy from foodstuff, regulate epithelial growth, prevent the colonization of pathogens, and provide many other benefits [43]. In addition, gut microbiota could induce potent regulatory effects on distant organs such as bone and brain. The gut microbiota regulates the bone matrix metabolism through multiple pathways including gut barrier permeability, nutrition absorption, metabolite production, immune response, and the endocrine system, which is referred to as the “microbiota-gut-bone axis” [44]. Microbial dysbiosis may affect bone metabolism by the gut microbiota in the trafficking of many metabolites, cytokines, and inflammatory cells to the bone marrow and influencing the overall inflammatory state of the patient, which is a common manifestation of the imbalance in “microbiota-gut-bone axis” [45]. For instance, the production of osteoclastogenic cytokine was always enhanced under estrogen deprivation, but this phenomenon fails to occur in germ-free (GF) mice. The colonization of the gut microbiota of CONV-R mice could restore the sensitivity of the skeletal system to sex steroid deficiency, which demonstrated the essential role of gut microbiota in the “microbiota-gut-bone axis” [46]. The utilization of probiotics or prebiotics has been proved to protect bone health by affecting SCFA production, inflammatory response, and other pathways [10]. Therefore, we investigated the changes in the diversity and composition of gut flora following GNS treatment by 16S rDNA sequencing. In terms of β-diversity, OVX + GNS group mice showed significant clustering separation from OVX group mice through PCoA, which indicated that GNS treatment markedly changed the biome structures. Furthermore, we performed LEfSe analysis between OVX + GNS and OVX groups to determine the predominant bacteria mediated through GNS treatment. GNS significantly decreased the abundance of *Alloprevotella* and *Bacteroidales*, which were known to favor the production of TMAO-related metabolites. Therefore, our results proved that GNS regulates osteoporosis-induced enteric dysbacteriosis by influencing the composition and structure of gut microbiota.

A matched case-control study found that high TMAO serum levels are related to an increased risk of hip fracture and osteoporosis in old people [47]. In addition, TMAO treatment promotes bone marrow mesenchymal stem cells (BMSCs) adipogenesis and attenuates osteogenesis, enhancing ROS release and production of pro-inflammatory cytokines such as IL-1β, IL-6, and TNF-α [16]. Given that the altered bacteria genera in the OVX + GNS group were associated with TMAO metabolism and TMAO concentration was increased in patients with osteoporosis, we evaluated TMAO levels between the OVX + GNS and OVX groups. TMAO-targeted metabolomics analysis revealed that GNS administration markedly reduced the production of microbiota-derived TMAO. In addition, FMT also alleviated microbiota-derived TMAO, further suggesting the key role of TMAO in GNS therapy. We subsequently performed a cytokine microarray assay and found that GNS significantly downregulated the level of pro-inflammatory cytokines IL-6, TNF-α, and G-CSF that manifest directly pro-osteoclastogenic activity. The reduction in pro-inflammatory cytokines may be due to the downregulation of TMAO. The specific mechanisms underlying the GNS-TMAO- pro-inflammatory cytokines need to be more investigated.

Meanwhile, there are many limitations in this study: (i) GNS led to significant increases in BV/TV, Tb.N, Tb.Th, and a reduction in Tb.Sp. Interestingly, there was no difference in BMD between the OVX and OVX + GNS groups, which may be due to the fact that the change of trabecular structure preceded the change of BMD. In addition, BMD means the bone mineral quantity per unit area that is a composite reflection of the bone mass in cortical and cancellous bone. Therefore, the insignificant alteration of bone mass in cortical bone may lead to no significant difference in BMD between the two groups. (ii) FMT only markedly increased BMD and Ct.Ar compared with the control group, and other indexes displayed no differences between the two groups. We think there is a certain loss of gut microbiota during FMT experiments, which leads to insignificant results in FMT groups. In addition, the gut-bone modulation axis requires more steps in osteoporosis and cannot be as direct as the gut microbiota regulation of colitis. Recent studies demonstrated that transient perturbations (such as OVX surgery) in gut homeostasis affect the long-term stability of the heterologous transplanted microbiota. The gut microbiota of the recipient mice is unable to maintain a homeostasis similar to that of the donor mice and may revert to the initial state before FMT [48]. Therefore, long-term attention should be paid to the stability of allogeneic intestinal flora after FMT and repeated transplantation should be carried out if necessary. (iii) Other regulatory pathways of gut microbiota on osteoporosis also need to be studied. For example, gut microbiota could directly regulate relevant signaling pathways through extracellular vesicles, thereby making effects on modulation of bone homeostasis. (iv) There may be other metabolic pathways such as short-chain fatty acids and bile acids in the gut-bone axis, which also need to be explored. (v) The specific bacterial genus that modulates TMAO metabolism in osteoporosis is uncertain and needs to be verified through any other meaningful experiments in vitro and in vivo. (vi) It is better to choose other types of nanoparticles to compare the performance with GNS, which is helpful to verify the good biocompatibility and anti-inflammatory activity of GNS. (vii) As mentioned above, osteoclasts are multinuclear giant cells derived from myeloid hematopoietic precursors, maintaining bone health with osteoblasts. The excessive activation of osteoclasts results in severe bone resorption and the loss of bone mass [49, 50]. The regulatory role of TMAO in osteoclastogenesis remains unclear, which may be the valuable point for further study on this topic. Taken altogether, we found that GNS treatment could ameliorate OVX-induced osteoporosis by remodeling the gut microbiota (Fig. 10). The potential protective mechanism was linked to the decreased level of pro-inflammatory cytokines mediated by the lower TMAO abundance. Our results demonstrated the role of GNS as an effective gut microbiota modulator and conferred a novel biochemical mechanism of GNS against osteoporosis. Although the specific mechanism in which GNS affects the intestinal flora needs more investigation, it still conserves an important basis for the future discovery and development of GNS-derived novel drugs.

## Electronic supplementary material

Below is the link to the electronic supplementary material.

Supplementary Material 1

## Data Availability

The data that support the findings of this study are available within the article and its supplementary materials. Raw data are available from the corresponding authors upon reasonable request.

## Abbreviations

ABX: Antibiotics
BM: Bone marrow
BMD: Bone mineral density
BMSCs: Bone marrow mesenchymal stem cells
BTMs: Bone turnover biomarkers
BV/TV: Trabecular bone volume fraction
Ct.Ar: Cortical bone area
Ct.Th: Cortical bone thickness
CTX-1: C-telopeptide of type I collagen
DECs: Differentially expressed cytokines
ELISA: Enzyme-linked immunosorbent assay
FCM: Flow cytometry
FM: Fecal microbiota
G-CSF: Granulocyte colony-stimulating factor
GNP: Gold nanoparticles
GNS: Gold-nanospheres
H&E: Hematoxylin and eosin
IL: Interleukin
LEfSe: Linear discriminant analysis effect size
NLRP3: Nucleotide-binding oligomerization domain-like receptor protein 3
OPLS-DA: Orthogonal projections to latent structures discriminate analysis
OTUs: Observed taxonomic units
OVX: Ovariectomy
PCA: Principle component analysis
PCoA: Principal coordinate analysis
PGF: Pseudo-germ-free
SEM: Scanning electron microscope
Tb.N: Trabecular number
Tb.Th: Trabecular thickness
Tb.Sp: Trabecular separation
TMA: Trimethylamine
TMAO: Trimethylamine N-oxide
TNF-α: Tumor necrosis factorα
Tregs: Regulatory T cells
TiO2: Titanium dioxide.
