# Supplementary material for: Gold-nanosphere mitigates osteoporosis through regulating TMAO metabolism in a gut microbiota-dependent manner
Source: J Nanobiotechnology. 2023 Apr 11;21:125. doi: 10.1186/s12951-023-01872-9 (PMC10088181; doi:10.1186/s12951-023-01872-9)
Supplement: Supplementary file 1 — Supplementary Material 1 [file 12951_2023_1872_MOESM1_ESM.docx]

**Gold-Nanosphere mitigates osteoporosis through regulating TMAO metabolism** **in a** **gut microbiota-dependent manner**

Yueqi Chen ^1*#^, Chuan Yang ^2#^, Qijie Dai ^1^, Jiulin Tan ^1^, Ce Dou ^1*^, Fei Luo ^1*^

^1^ Department of Orthopedics, Southwest Hospital, Third Military Medical University (Army Medical University), Chongqing, People’s Republic of China

^2^ Department of Biomedical Materials Science, Third Military Medical University (Army Medical University), Chongqing, People’s Republic of China

^#^ Yueqi Chen and Chuan Yang contributed equally to this work.

**^*^** **Correspondence**:

Dr. Yueqi Chen, Department of Orthopedics, Southwest Hospital, Third Military Medical University (Army Medical University), Chongqing, People’s Republic of China

Email: chenyueqi1012@sina.com

Dr. Ce Dou, Department of Orthopedics, Southwest Hospital, Third Military Medical University (Army Medical University), Chongqing, People’s Republic of China

Email: lance.douce@gmail.com

Prof. Fei Luo, Department of Orthopedics, Southwest Hospital, Third Military Medical University (Army Medical University), Chongqing, People’s Republic of China

Email: [luofly1009@hotmail.com](mailto:luofly1009@hotmail.com)

**Supplementary Materials and Methods**

**Materials**

| **Reagent** | **Source** | **Code number** | **Country** |
| --- | --- | --- | --- |
| GNS | Wuhan MICE Biotechnology Co. Ltd | None | China |
| vancomycin | MedChemExpress | [HY-B0671](https://www.medchemexpress.cn/vancomycin.html) | United States |
| neomycin | MedChemExpress | [HY-B0470](https://www.medchemexpress.cn/Neomycin-sulfate.html) | United States |
| metronidazole | MedChemExpress | [HY-B0318](https://www.medchemexpress.cn/Metronidazole.html) | United States |
| ampicillin | MedChemExpress | [HY-B0522](https://www.medchemexpress.cn/Ampicillin.html) | United States |
| CTX-1 ELISA Kits | Novus | NBP2-69074 | United States |

**Synthesis of GNS**

The first step is the synthesis of gold seeds. Trisodium citrate was used as a reducing agent and protective agent, and chloroauric acid was used as a gold precursor. The size of the seeds was controlled by adjusting the amount of reducing agent. In the second step, the seeds were taken as the core, trisodium citrate as the protective agent, and ascorbic acid as the reducing agent. The GNS with different particle sizes were obtained by adjusting the amount of chloroauric acid.

**Fecal genomic DNA extraction, PCR amplification and 16S rDNA sequencing**

The total DNA from the two groups was extracted using the E.Z.N.A. ®Stool DNA Kit (D4015, Omega, Inc., USA) in accordance with the manufacturer’s instructions. The reagent has been proved to be effective for the DNA preparation of most bacteria that could uncover DNA from trace amounts of sample. Nuclear-free water was used for blank. The measurement in PCR was performed by LC-Bio Technology Co., Ltd (Hang Zhou, China).

Specific barcodes were used to label the 5' end of the primers in each sample, and then the universal primers were sequenced. The total volume of reaction mixture used to perform PCR amplification was 25 μl consisting of 25 ng of template DNA, 12.5 μl PCR Premix, 2.5 μl of each primer, and PCR-grade water was used to adjust the volume. The PCR conditions to amplify the prokaryotic 16S fragments were as follows: an initial denaturation at 98 ℃ for 30 seconds; 32cycles of denaturation at 98 ℃ for 10 seconds, annealing at 54℃ for 30 seconds, and extension at 72 ℃ for 45 seconds; and then final extension at 72 ℃ for 10 minutes. Then we used 2% agarose gel electrophoresis to confirm the PCR products. The ultrapure water was used in the whole DNA extraction process to rule out the possibility of false-positive PCR results. AMPure XT beads (Beckman Coulter Genomics, Danvers, MA, USA) were used to sublimate the PCR products, which were subsequently quantified by Qubit (Invitrogen, USA). The amplicon pools were prepared for sequencing. The size of the amplicon library was assessed on Agilent 2100 Bioanalyzer (Agilent, USA) and the quantity was assessed with the Library Quantification Kit for Illumina (Kapa Biosciences, Woburn, MA, USA). Finally, we used the NovaSeq PE250 platform to perform sequencing of the libraries.

According to the unique barcodes, the paired-end reads were assigned to the sample, and the barcodes and primer sequences introduced by the construction of the library were removed. Paired-end reads were merged using FLASH. According to fqtrim (v0.94), the raw read data was qualitatively filtered under specific filtering conditions to obtain a high-quality clean label. The chimeric sequences were filtered using Vsearch software (v2.3.4). Then we use DADA2 to perform dereplication to obtain feature table and feature sequence. Alpha diversity and beta diversity were calculated by normalizing them to the same random sequence. Subsequently, according to SILVA (release 132) classifier, the relative abundance of each sample was used to normalize the feature abundance. Alpha diversity included Chao1, Observed species, Shannon, and Simpson, which were calculated with QIIME2. Beta diversity was also calculated by QIIME2, and the graphs were drawn by the R package. Blast was used for sequence alignment, and the feature sequences were annotated with the SILVA database for each representative sequence. Other diagrams were implemented using the R package (v3.5.2).

**Fecal TMAO metabolomics quantitative analysis**

50 mg feces of each sample were mixed with 1 ml extraction solution that consisted of acetonitrile, methanol and water (2:2:1, with isotopically-labeled internal standard mixture) in an EP tube. The mixture was vortexed for at least 30s, homogenized at 35Hz for 4 min and sonicated for 5min in ice-water bath. The above processes were repeated 3 times. Then the samples were incubated for 1h at -40°C and centrifuged at 12000rpm for 15min at 4°C. The supernatant was transferred to a fresh glass and the supernatant of each sample was mixed in equal amounts to prepare a quality control (QC) sample. LC-MS/MS analyses were performed using a UHPLC system (Vanquish, Thermo Fisher Scientific) with a UPLC BEH Amide column (2.1 mm × 100 mm, 1.7 μm) coupled to Q Exactive HFX mass spectrometer (Orbitrap MS, Thermo). Then we used ProteoWizard to convert the raw data into the mzXML format and performed peak detection, extraction, alignment, and integration using an in-house program that was developed by R and based on XCMS. The metabolite annotation was performed by an in-house MS2 database (Biotree DB). All these procedures were performed and supported at Biotree Biomedical Technology (Shanghai, China). Then the data were trimmed using Compound Discoverer 2.1 (Thermo Fisher Scientific, Waltham, MA, United States). The principal component analysis (PCA) and orthogonal projections to latent structures discriminate analysis (OPLS-DA) were performed in the SIMCA16.0.2 software package (Sartorius Stedim Data Analytics AB, Umea, Sweden). The metabolites with VIP > 1.0 and p < 0.05 (Student’s t-test) were considered to possess significant differences.


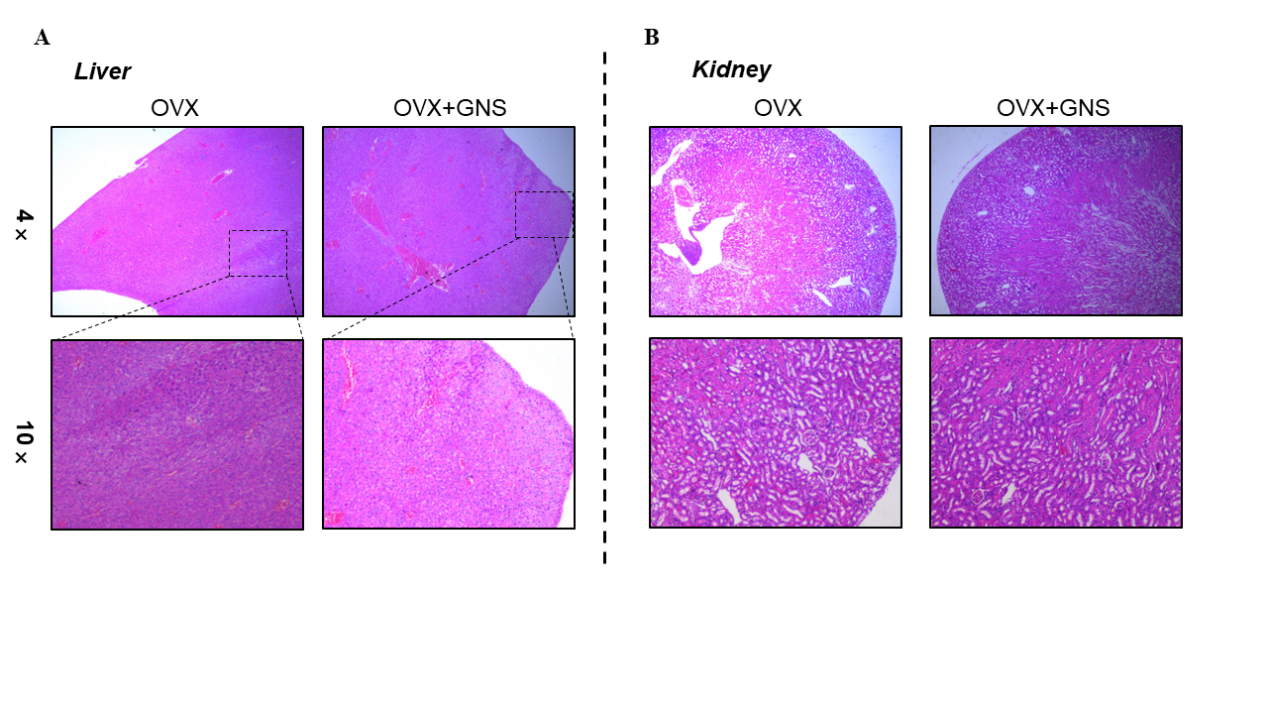


**Supplementary Figure S1.** GNS displayed no liver and kidney toxicity in mice.

(A) Representative pictures of liver with H&E staining in OVX and OVX +GNS groups.

(B) Representative pictures of kidney with H&E staining in OVX and OVX +GNS groups.


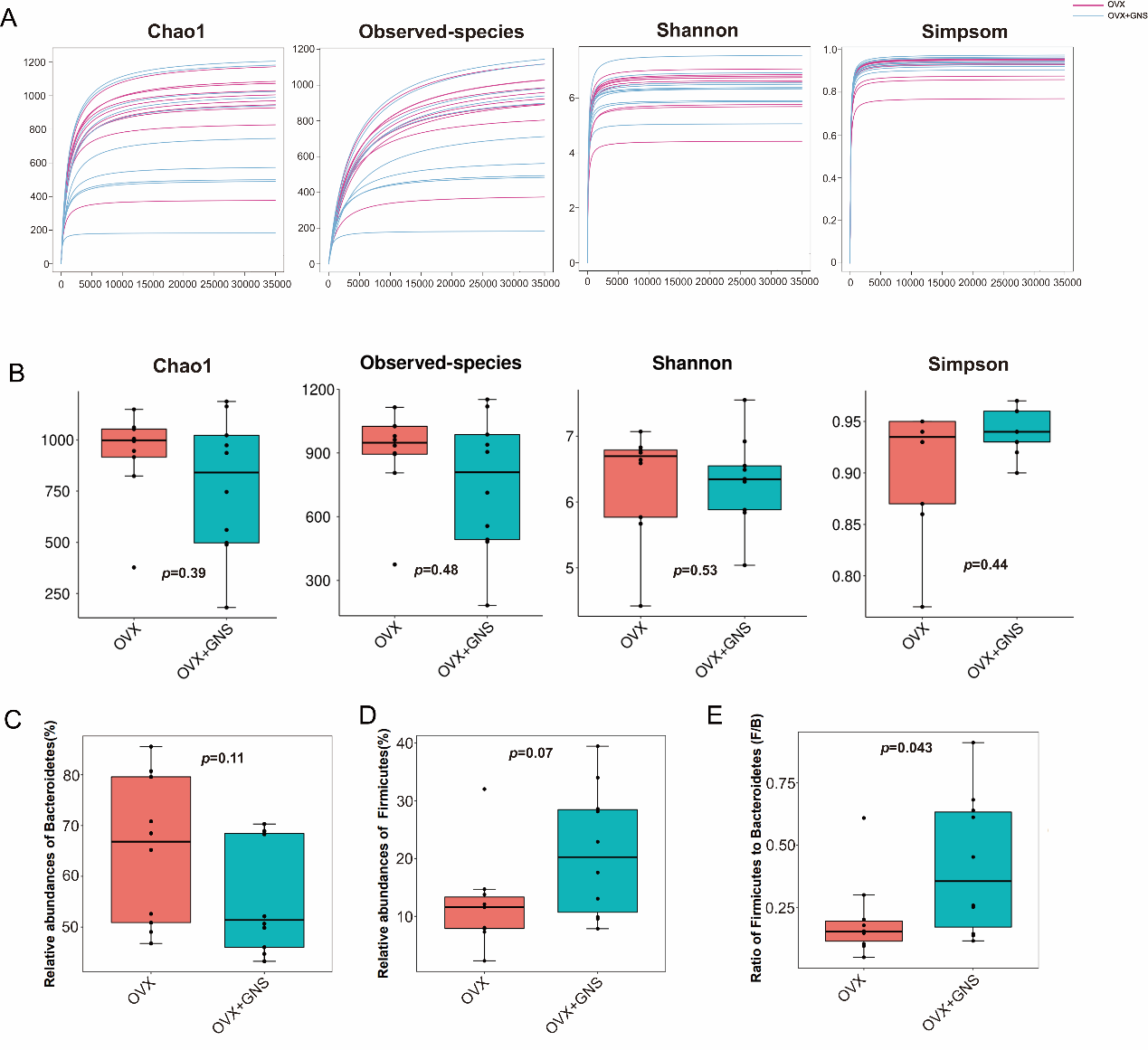


**Supplementary Figure S2.** The analyses of alpha diversity and the predominating bacterial phyla at phylum taxonomic level.

(A) Rarefaction curve based on alpha diversity.

(B) Alpha diversity boxplot including observed species, Shannon, Simpson and Chao1.

(C) The relative abundances of *Bacteroidetes*.

(D) The relative abundances of *Firmicutes*.

(E) The ratio of *Firmicutes* to *Bacteroidetes* (F/B).


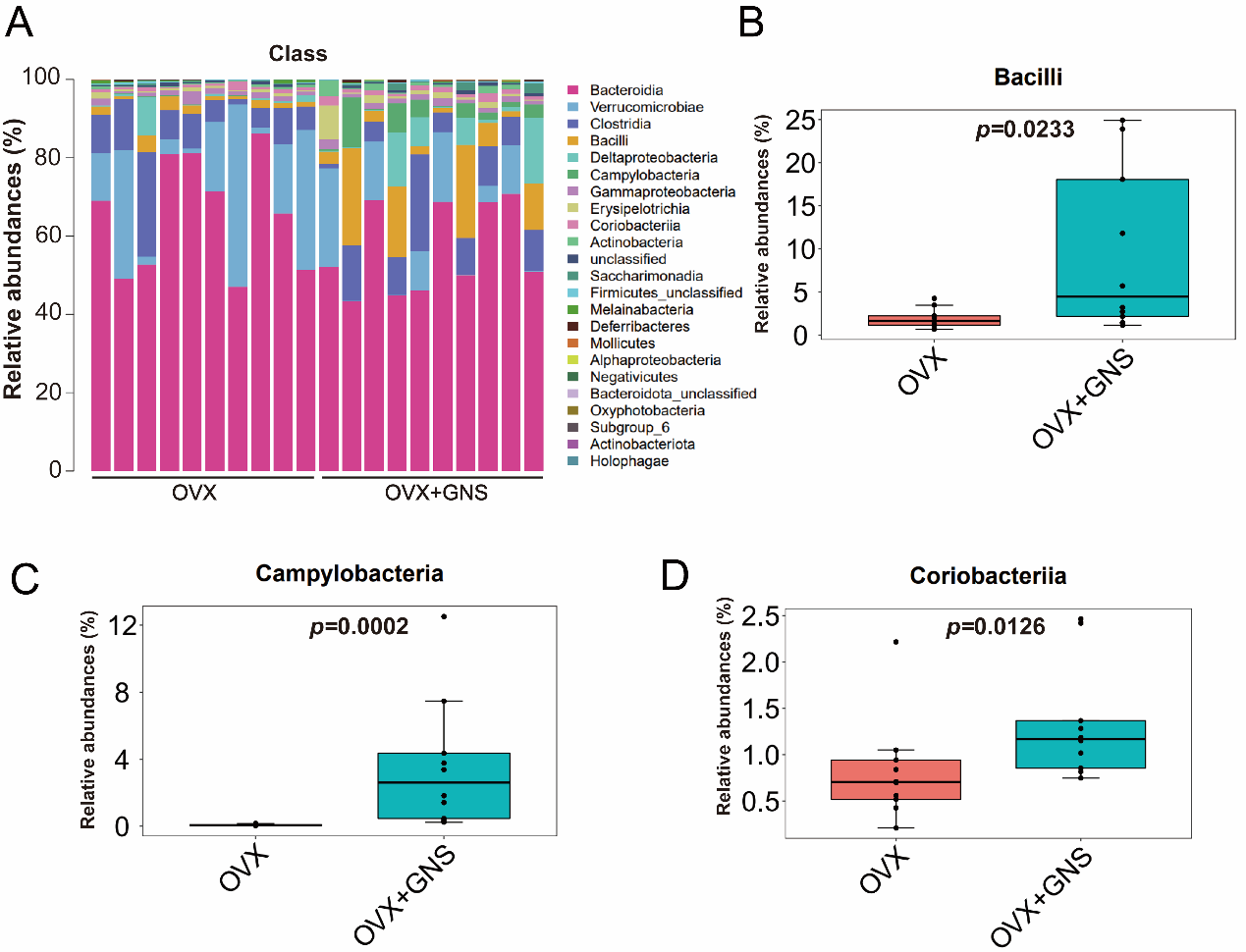


**Supplementary Figure S3.** The composition and diversity of gut microbiota at the class taxonomic level.

(A) Bar graphs of the bacteria at the class taxonomic level between OVX and OVX+GNS groups. The relative abundance of each sample was plotted.

(B) The relative abundances of *Bacilli* in two groups.

(C) The relative abundances of *Campylobacteria* in two groups.

(D) The relative abundances of *Coriobacteriia* in two groups.


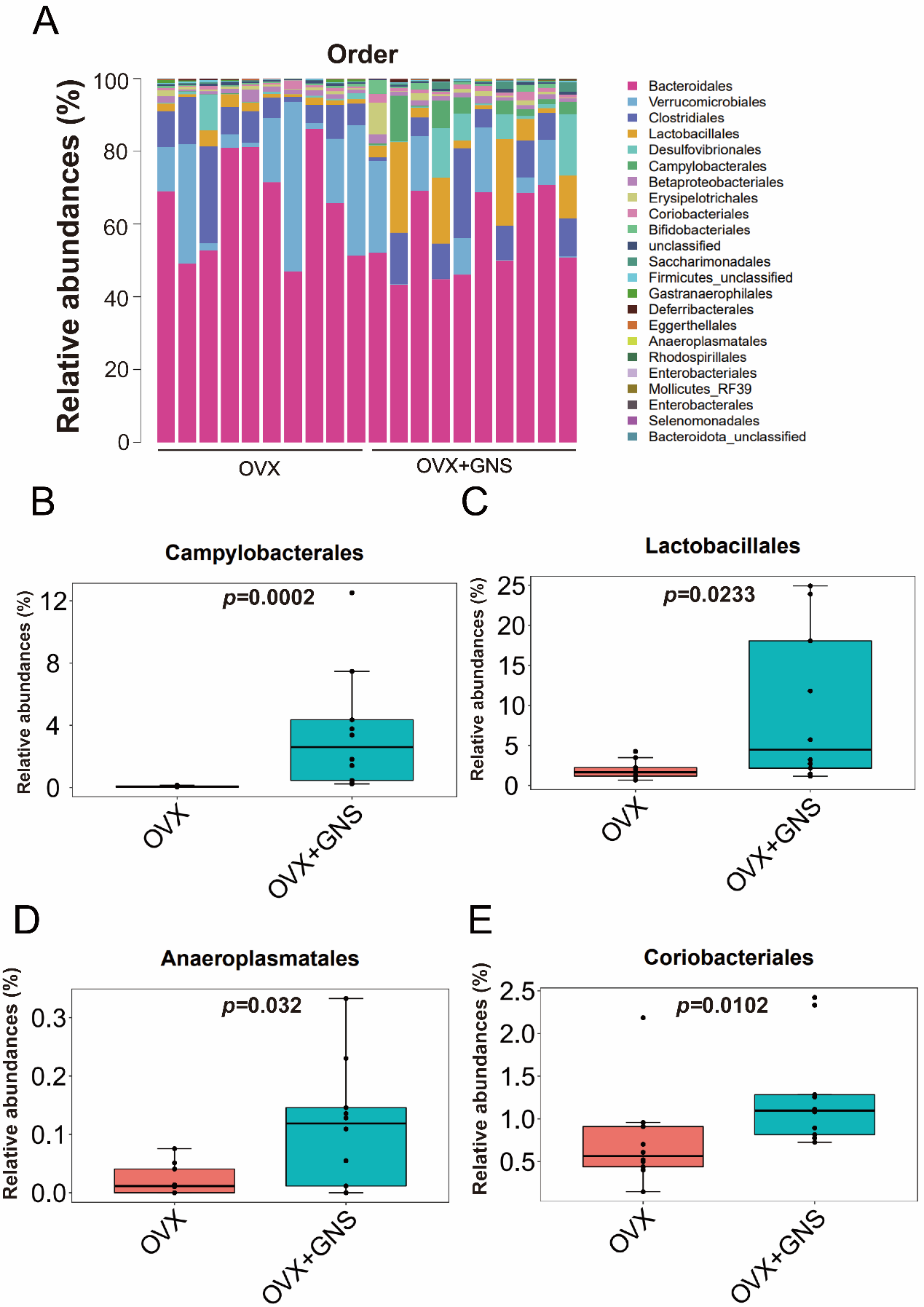


**Supplementary Figure S4.** The composition and diversity of gut microbiota at the order taxonomic level.

(A) Bar graphs of the bacteria at order taxonomic level between OVX and OVX+GNS groups. The relative abundance of each sample was plotted.

(B) The relative abundances of *Campylobacterales* in two groups.

(C) The relative abundances of *Lactobacillales* in two groups.

(D) The relative abundances of *Anaeroplasmatales* in two groups.

(E) The relative abundances of *Coriobacteriales* in two groups.


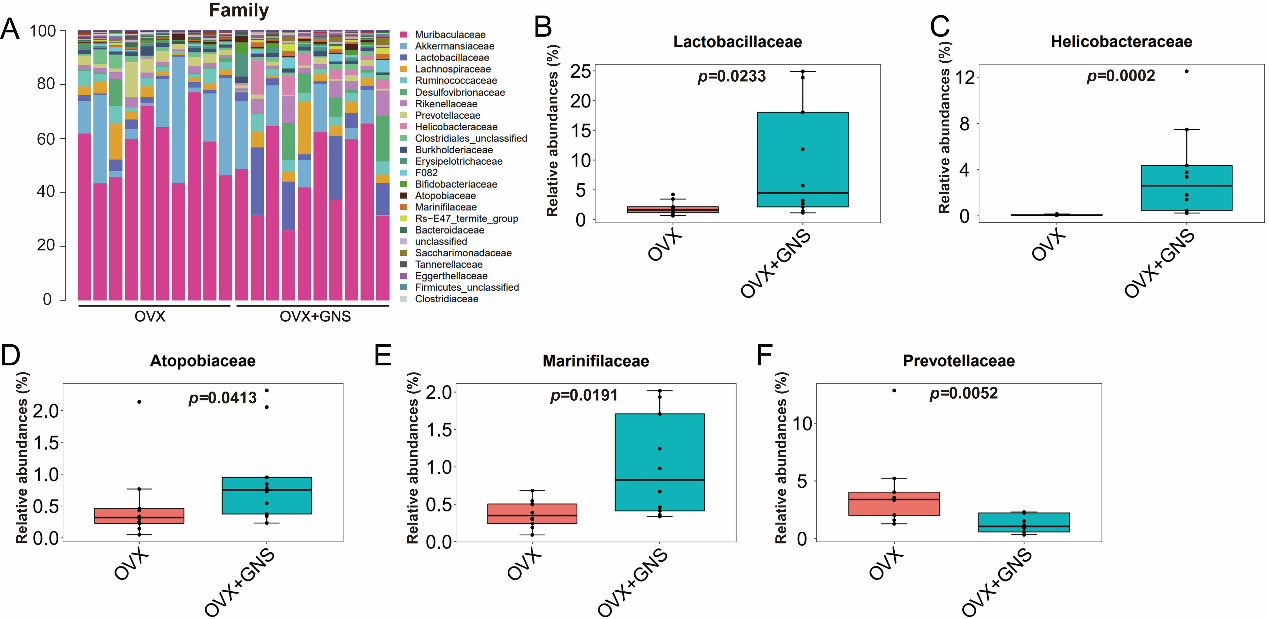


**Supplementary Figure S5.** The composition and diversity of gut microbiota at the family taxonomic level.

(A) Bar graphs of the bacteria at the family taxonomic level between OVX and OVX+GNS groups. The relative abundance of each sample was plotted.

(B) The relative abundances of *Lactobacillaceae* in two groups.

(C) The relative abundances of *Helicobacteraceae* in two groups.

(D) The relative abundances of *Atopobiaceae* in two groups.

(E) The relative abundances of *Marinifilaceae* in two groups.

(F) The relative abundances of *Prevotellaceae* in two groups.


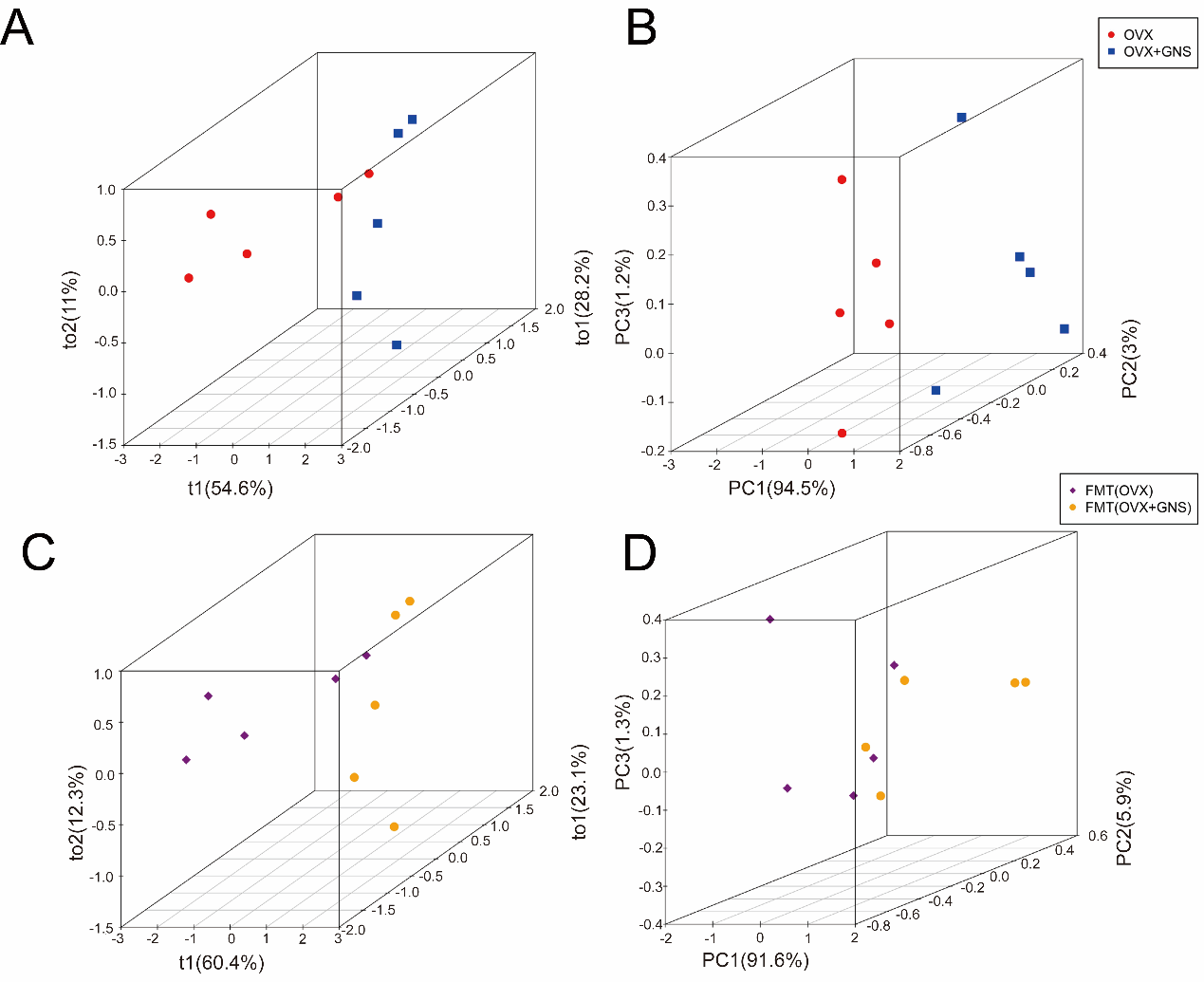


**Supplementary Figure S6.** The OPLS-DA and PCA score plot of TMAO.

(A) OPLS-DA score chart of TMAO between OVX and OVX+GNS groups.

(B) PCA score chart of TMAO between OVX and OVX+GNS groups.

(C) OPLS-DA score plot of TMAO between FMT(OVX) and FMT (OVX+GNS) groups.

(D) PCA score plot of TMAO between FMT(OVX) and FMT(OVX+GNS) groups.
